# Intermittent hypoxia induces reversible epigenetic age acceleration in old mice

**DOI:** 10.1038/s41514-026-00425-2

**Published:** 2026-06-23

**Authors:** Stefano Donega, Ake T. Lu, Amin Haghani, Markus Horvath, Qi Yan, Mirko Baranzini, Maxine Thea Escote, Martina Rossi, Rolando J. Hernandez, Ross A. McDevitt, Kenneth W. Fishbein, Steve Horvath, Luigi Ferrucci

**Affiliations:** 1 Longitudinal Studies Section, Translational Gerontology Branch, National Institute on Aging, National Institutes of Health, USA, Baltimore, MD USA; 2 Epigenetic Clock Development Foundation, Torrance, CA USA; 3 Altos Labs, San Diego Institute, California, CA USA; 4 Comparative Medicine Section, National Institute on Aging, National Institutes of Health, USA, Baltimore, MD USA; 5 MRI Core Facility for Translational Research, National Institute on Aging, National Institutes of Health, Baltimore, MD USA; 6 Department of Medicine, Division of Geriatrics, David Geffen School of Medicine, University of California, Los Angeles, CA USA

**Keywords:** Developmental biology, Genetics, Molecular biology

## Abstract

Epigenetic mechanisms are considered adaptive regulators of gene expression, yet mechanisms driving aging-associated DNA methylation remain unclear. Prior work hinted that epigenetic aging might reflect a response to oxygen availability, with age‑differential methylation in immune cells enriched near binding sites for hypoxia‑responsive factors ARNT and REST. To test this hypothesis, we exposed adult (11 months) and old (23 months) mice to 1 month of intermittent hypoxia (IH) followed by normoxic recovery. IH induced epigenetic age acceleration in lungs, spleen, and heart in old mice only. This acceleration reversed upon return to normoxia. Reversible shifts were enriched at bivalent domains and PRC2 targets, indicating oxygen-sensitive chromatin remodeling. Human translational validation in young adults at high altitude (5260 m) confirmed rapid, conserved epigenetic aging. Our findings establish oxygen availability as a primary, conserved modulator of epigenetic aging across tissues and species, showing that oxygen fluctuations are a potent, reversible driver of epigenetic aging.

## Introduction

Epigenetic mechanisms, particularly DNA methylation, can modulate gene expression without altering the underlying DNA sequence^1^. The transcriptional program activated by epigenetic changes serves as a critical interface between the genome and the environment, functioning as an adaptive response to stressors^2,3^. It is well-established that specific CpG sites (sites where a cytosine nucleotide is followed by a guanine nucleotide) exhibit predictable and widespread changes in methylation status with aging. The weighted average of DNA methylation at specific CpG sites forms the basis of the epigenetic clock, a metric that approximates an organism’s chronological age. A subset of age-related DNA methylation changes is conserved across multiple tissue types and immune cell lineages^4–6^. The discrepancy between an individual’s epigenetic age and their chronological age is a significant predictor of adverse health outcomes^7,8^. The development of pan-tissue and even pan-mammalian clocks suggests that a fundamental, conserved biological process may drive a portion of the epigenetic aging signal, although the full mechanisms remain to be elucidated^9–11^.

To shed light on the biological mechanisms underlying age-associated DNA methylation alterations, we previously examined methylation profiles of DNA extracted from cell-sorted circulating immune cells obtained from healthy individuals spanning a broad age range^6^. Across multiple cell types, there was striking enrichment of age-related CpGs near the binding motifs for the transcription factors ARNT (also known as HIF1B) and REST, which are central regulators of the cellular response to hypoxia^12–16^. Based on these findings, we hypothesized that some age-related DNA methylation changes are driven by and possibly adaptive to fluctuations in oxygen availability across tissues^17,18^. This theory is biologically plausible, as aging is associated with physiological changes that may cause pockets of relative tissue hypoxia. For example, the age-related dysfunction of the microcirculatory mechanisms responsible for fine-tuning tissue perfusion and oxygen transport may cause areas of hypoxia, especially in the most energy-demanding tissues^19,20^. Furthermore, chronic inflammation and fibrosis, both of which are common in older individuals, can induce tissue hypoxia^21,22^. Consistent with the idea of hypoxia driving epigenetic changes, prior exposure to hypoxia has been shown to induce an adaptive epigenetic response, leading to greater cellular resilience and longer survival after subsequent hypoxic challenges^17,23,24^.

Environmental stimuli can induce epigenetic changes that contribute to adaptive, neutral, or maladaptive responses depending on dose, duration, and tissue context. One of these stimuli is hypoxia, since it can activate adaptive pathways associated with cellular acclimation and hormesis^25^, whereas more severe or prolonged hypoxia can trigger oxidative stress, inflammation, and maladaptive remodeling^26^. Intermittent hypoxia conditioning paradigms likewise show protocol‑dependent outcomes, and even hyperbaric oxygen therapy (which increases oxygen availability) can carry adverse effects at certain doses^27^.

In this study, we developed an in vivo strategy to directly test whether a hypoxia regimen accelerates epigenetic aging and to assess the potential reversibility of these effects. We exposed adult and old female mice to a rigorous and controlled protocol of intermittent hypoxia (IH) for 1 month, followed by a one-month recovery period. To evaluate the impact of oxygen fluctuations, we analyzed DNA methylation patterns and epigenetic age across multiple tissues, determining both the extent of age acceleration after hypoxia and the degree of reversibility after recovery. We focused on the lung, heart, and spleen because they represent three physiologically distinct interfaces with hypoxia. The lung is the primary site of gas exchange and directly experiences oscillatory oxygen fluctuations; the heart is highly oxygen dependent and sensitive to hypoxia–reoxygenation stress; and the spleen is enriched for immune cell populations in which we previously identified hypoxia-associated transcription factors binding sites close to the aging CpG methylation signatures. To determine whether our observations in mice also apply to humans, we analyzed publicly available blood methylation data from young individuals who were exposed to hypobaric low oxygen during high-altitude (5260 m) acclimation at Mt. Chacaltaya, Bolivia.

## Results

### Experimental design and HIF1A assessment

**Fig. 1:**
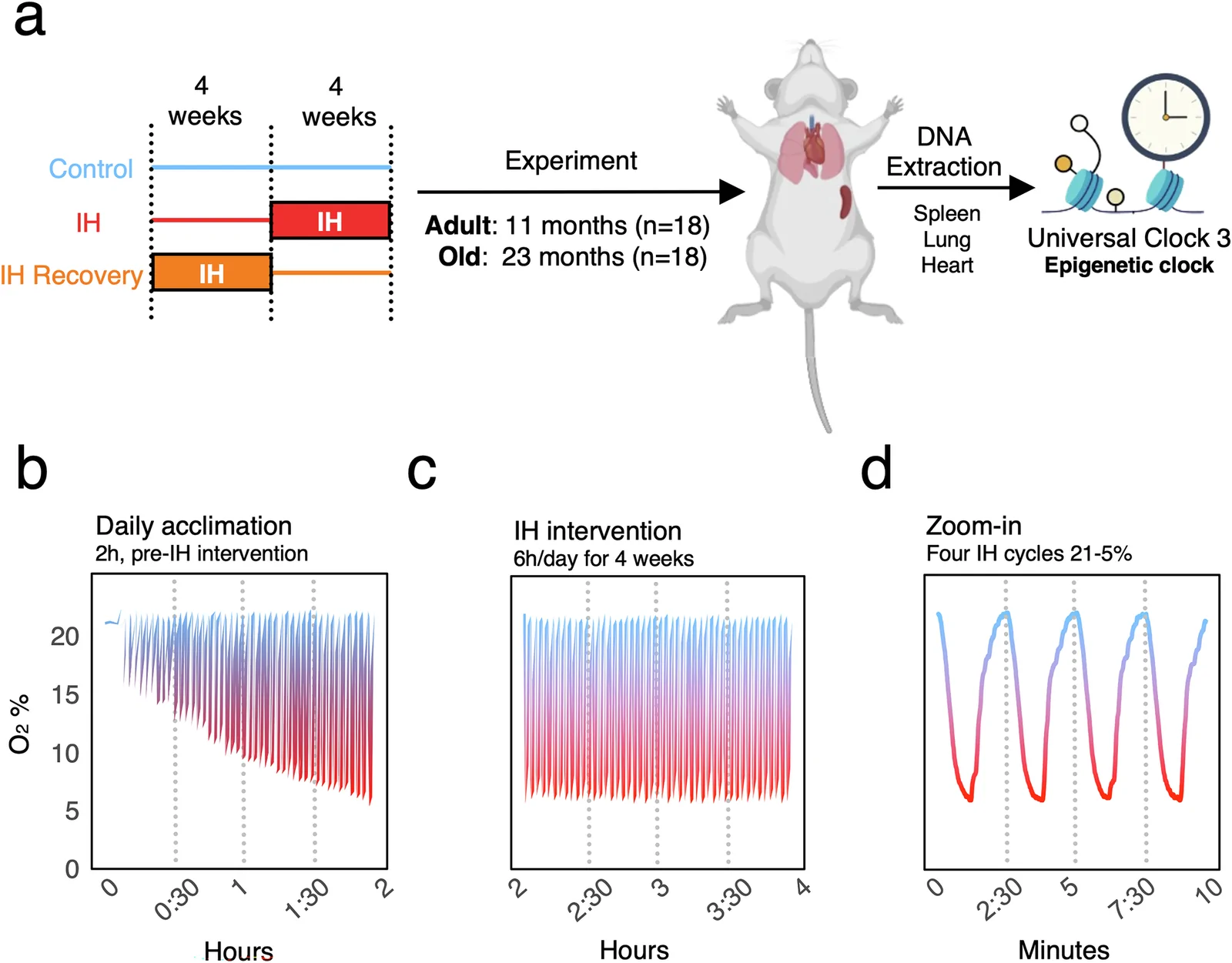
Experimental design and intermittent hypoxia (IH) exposure. **a** Experimental design: Female C57BL/6 mice (*n* = 36) were divided into two age groups: adult (~11 months) and old (~23 months) and randomly assigned to one of three conditions: Control (maintained under normoxia), IH (1 month of normoxia followed by 1 month of IH), or IH Recovery (1 month of IH followed by 1 month recovery in normoxia). All groups contained *n* = 6 mice. **b** IH daily acclimation (2 h, pre-intervention): schematic of the daily 2-h acclimation period with stepwise oxygen reduction. **c** IH schedule: schematic representation of a 2-h interval (immediately after daily acclimation) to represent the 6-h daily intervention over 4 weeks. **d** Oxygen cycle profile: A representative trace of the 2 min and 30 s cycles oscillating between 21 and 5% O₂ during an 8-h daily session. Although oxygen probes were not placed inside cages during the animal experiment, independent validation tests confirmed that the oscillatory oxygen profile is uniform across cage positions, without relevant spatial variation.

Details of the experimental design and technical procedures are provided in the Methods section. Briefly, a total of *n* = 36 female mice (~11 months, adult; ~23 months, old; *n* = 6 per group for all age and exposure conditions) were assigned to one of three conditions: control, 1 month of intermittent hypoxia (IH), or 1 month of IH followed by 1 month of recovery (Fig. 1a). Based on hierarchical clustering analysis, six samples from the old group (one in heart, two in spleen, and three in lung) showed low quality in their DNA methylation arrays and were removed from subsequent analysis. This yielded a total of 102 samples across 35 mice remaining in our study. Prior to the 1-month intermittent hypoxia exposure, mice underwent a 5-day acclimatization protocol using an IH system where oxygen concentration was progressively reduced to ensure survival.

The protocol was designed to gradually lower the minimum oxygen level from 15 to 6%, using the previous day’s lowest concentration as the starting point for the next reduction. Specifically, Day 1 consisted of 8 h cycling between 21 and 15% oxygen; Day 2 began with 2 h at 15% followed by 6 h at 10%; Day 3 involved a progressive decline with 1 hour at 15%, 1 h at 10%, 3 h at 9%, and 3 h at 8%; Day 4 consisted of 2 h at 8% followed by 6 h at 7%; and finally, on Day 5, the oxygen concentration reached a minimum of 6% (Fig. 1b). Next, mice were exposed to IH for 8 h/day, and each daily session consisted of a 2-h transition phase followed by 6 h of 2:30 min cycles oscillating between 21 and 5% O₂, for 30 days (Fig. 1c, d). Notably, over the 2-month study period, no behavioral differences or significant changes in body weight were observed among the three intervention groups (data not shown).

HIF1A is a well-established marker of hypoxia as it serves as the initial trigger of the molecular cascade that activates the hypoxia response. In the control group (no IH intervention, *n* = 4 per age group), lung tissue from older mice exhibited significantly higher HIF1A levels compared to adult mice (*t*-test, *p* = 0.03; Supplementary Fig. 1a, with uncropped gels in Supplementary Fig. 2). Adult mice showed no evident or significant differences in HIF1A expression across conditions (ANOVA *p* = 0.54; Supplementary Fig. 1b, left). In older mice, a trend toward increased HIF1A levels was observed in the IH group relative to controls, although this did not reach statistical significance (ANOVA *p* = 0.34; Supplementary Fig. 1b, right). To complement these Western blot findings, we quantified HIF1A protein levels by ELISA in a larger cohort of mice (*n* = 23 for lungs and *n* = 21 for spleen, see Methods). In lung tissue, neither Age, Condition, nor their interaction significantly influenced HIF1A concentrations (mixed‑effects model: Age *p* = 0.16, Condition *p* = 0.36, Age × Condition *p* = 0.24; Supplementary Fig. 1c). Similarly, spleen tissue showed no significant effects of Condition or Age × Condition, although a suggestive trend toward higher HIF1A levels with age was observed (Age *p* = 0.06; Supplementary Fig. 1d).

### IH accelerates the lung, spleen, and heart epigenetic clocks

The primary aim of this study was to determine whether IH accelerates epigenetic aging in multiple tissues and to evaluate the reversibility of these effects. Epigenetic age was quantified using the most recent pan-mammalian, multi-tissue predictor, the Universal Clock 3. Confirmatory analyses were also performed using Universal Clock 2 and mouse pan-tissue DNAmAge clock.

**Fig. 2:**
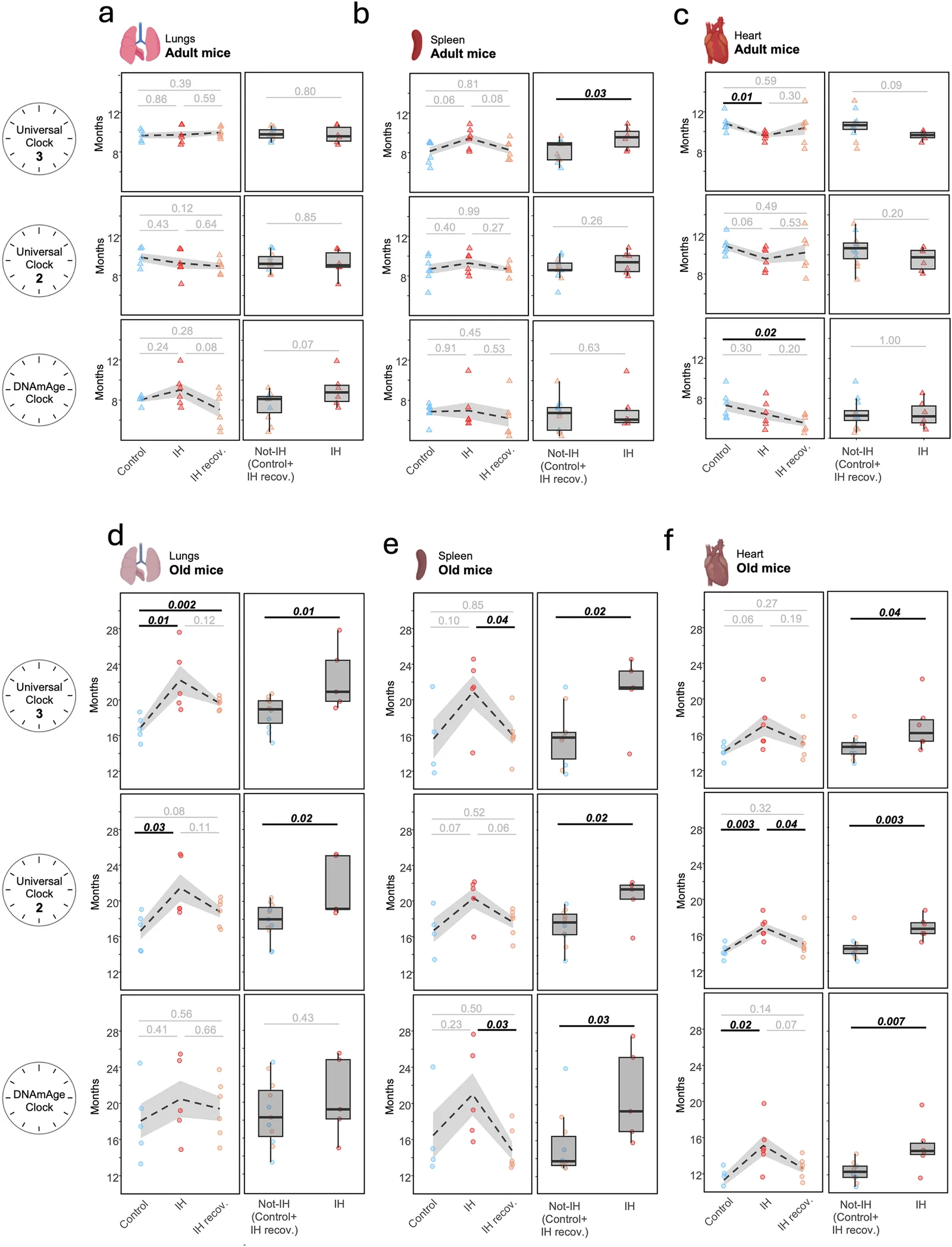
Epigenetic aging and reversible DNA-methylation changes in adult and old mice exposed to intermittent hypoxia (IH). Data were shown for three clocks: Universal Clock 3 (top row), Universal Clock 2 (middle row), and mouse DNAmAge (bottom row), in two age groups (Adult: panels (**a**–**c**); Old: panels (**d**–**f**)) across lung, spleen, and heart. For each panel, three treatment groups are displayed: Control, IH, and IH + recovery (IH_Rec). Dots represent individual mice (*n* = 6 per group); dotted lines connect group means to illustrate trends only (not longitudinal pairing). Triangles represent data points for adult mice, while circles represent older mice. To assess the overall IH effect, Control and IH_Rec were combined into a “Not-IH” group and compared with IH using boxplots placed to the right of the trajectories. Statistical testing used Student’s *t*-tests assuming equal variances for pairwise comparisons among condition groups and for IH vs Not-IH. X-axes represent condition group (Control, IH, IH_Rec, or Not-IH), while y-axes represent Epigenetic age (expressed in months). Dotted lines show group means; gray ribbons indicate mean ± standard error (SE).

As shown in Fig. 2, in adult animals, intermittent hypoxia (IH) had no significant impact on epigenetic age. In lung and heart tissues, all three clocks showed similar epigenetic ages at baseline, IH, and recovery. Nominally a significant finding was found in the spleen, where Universal Clock 3 detected a difference between IH and non-IH groups (*p* = 0.03). In the heart, the same clock showed a reduction in the IH group relative to control; (*p* = 0.01), this was not sustained, and with the DNAmAge clock the IH-recovery group was instead significantly reduced relative to control (*p* = 0.02). As expected in the absence of a true effect, these scattered nominal results were not consistent across clocks.

In contrast, older mice exhibited a clear and robust response: 1 month of IH caused pronounced acceleration of epigenetic age across all clocks and tissues, which were either statistically significant or showed clear trends. The only exception was lung tissue measured with the mouse pan-tissue, DNAmAge clock, which showed no statistical difference. In all other comparisons, IH exposure significantly increased epigenetic age: 4.92 months in the spleen, 5.28 months in the lung, and 2.88 months in the heart (Table 1). After 1 month of normoxic recovery, the epigenetic age of the spleen came back to baseline, with negligible residual acceleration (0.12 months, equivalent to only a few days). The epigenetic age of the heart also nearly returned to baseline, with only modest residual acceleration (0.96 months). In contrast, we observed incomplete recovery of epigenetic age in the lungs, with persistent acceleration of 2.76 months, suggesting partial but not full reversal of IH-induced accelerated aging. Further analyses were aimed at comparing the epigenetic age of mice after IH against mice not recently exposed to IH, namely those evaluated at baseline and those evaluated after a month of recovery. In adult mice, no significant differences were observed between non-IH and IH groups, except for Universal Clock 3 in the spleen. In contrast, all three clocks revealed significant differences in all three tissues of older mice, with the sole exception of DNAmAge in the lungs. These striking findings demonstrate that the IH intervention elicited a unique and clear effect in the old mouse cohort.

**Table 1:** IH-dependent acceleration of epigenetic age across tissues using Universal Clock 3

Tissue	Age	Condition	Age (months)	Univ. Clock 3 (months)	Delta Age (months, to control)	Univ. Clock 3 (years)	Delta Age (years, to control)
**Lung**	**Adult**	Control	10.8	9.7	-	0.81	-
		IH	10.8	9.7	0	0.81	0
		IH Rec.	10.8	10	0.24	0.83	0.02
	**Old**	Control	22.7	16.9	-	1.41	-
		IH	23.4	22.2	***5.28***	1.85	0.44
		IH Rec.	22.8	19.7	***2.76***	1.64	0.23
**Spleen**	**Adult**	Control	10.8	8.2	-	0.68	-
		IH	10.8	9.5	1.32	0.79	0.11
		IH Rec.	10.8	8.3	0.12	0.69	0.01
	**Old**	Control	22.4	16	-	1.33	-
		IH	23.4	20.9	**4.92**	1.74	0.41
		IH Rec.	22.8	16.1	**0.12**	1.34	0.01
**Heart**	**Adult**	Control	10.8	10.8	-	0.9	-
		IH	10.8	9.6	−1.2	0.8	-0.1
		IH Rec.	10.8	10.3	−0.48	0.86	-0.04
	**Old**	Control	22.7	14.2	-	1.18	-
		IH	23.5	17	**2.88**	1.42	0.24
		IH Rec.	22.8	15.1	**0.96**	1.26	0.08

For each tissue and age group (Adult, Old), the table lists the chronological mean age and the DNAm age estimated by Universal Clock 3. Within each tissue and age group, epigenetic age acceleration (Delta Age) is shown as the difference in values computed relative to the matched Control. Universal Clock 3 outputs are shown in months and years. Univ denotes Universal.

Results obtained with Universal Clock 3 and Universal Clock 2 were highly consistent with those obtained using DNAmAge, particularly in spleen and heart tissue of adult mice. We attribute small differences to the fact that the DNAmAge clock was trained exclusively on pan-tissue mouse data (385 CpGs)^28^, whereas the CpGs incorporated in the two Universal Clocks are cross-species predictors optimized for pan-mammalian application across 185 mammalian species (816 CpG sites for Universal Clock 2 and 760 for Universal Clock 3). The Universal Clocks have the potential to capture the changes from the genomic regions conserved among mammals. To better compare the three clocks, we also performed a pairwise correlation between the three clocks in the three organs, each for the three conditions (Supplementary Fig. 3). Universal Clock 3 and 2 were highly concordant: In adult mice the correlation measured using Pearson’s method ranged between 0.60 and 0.93 (with two exceptions for lung control and heart IH with 0.20 and 0.47, respectively), while in old mice most of the correlations were around 0.93 up to 0.98. The concordance between the Universal Clocks and the mouse-specific DNAmAge clock was notably more varied, particularly within the adult cohort. For example, correlations between Universal Clock 2 and DNAmAge in adult spleen samples ranged from 0.33 to 0.86 (Supplementary Fig. 3a). This correlation strength weakened further in the lung and heart groups; notably, we observed negative correlations in both adult lung controls (*r* = −0.33) and heart controls (*r* = −0.49). These data suggest that hypoxia-associated epigenetic aging transitions toward a more homogenous state in older mice. Conversely, adult mice exhibit a more heterogeneous epigenetic landscape, as reflected by the marked discordance in clock correlations.

### Epigenome-wide association studies (EWAS) reveal fully reversible age-specific CpG subsets across tissues

**Fig. 3:**
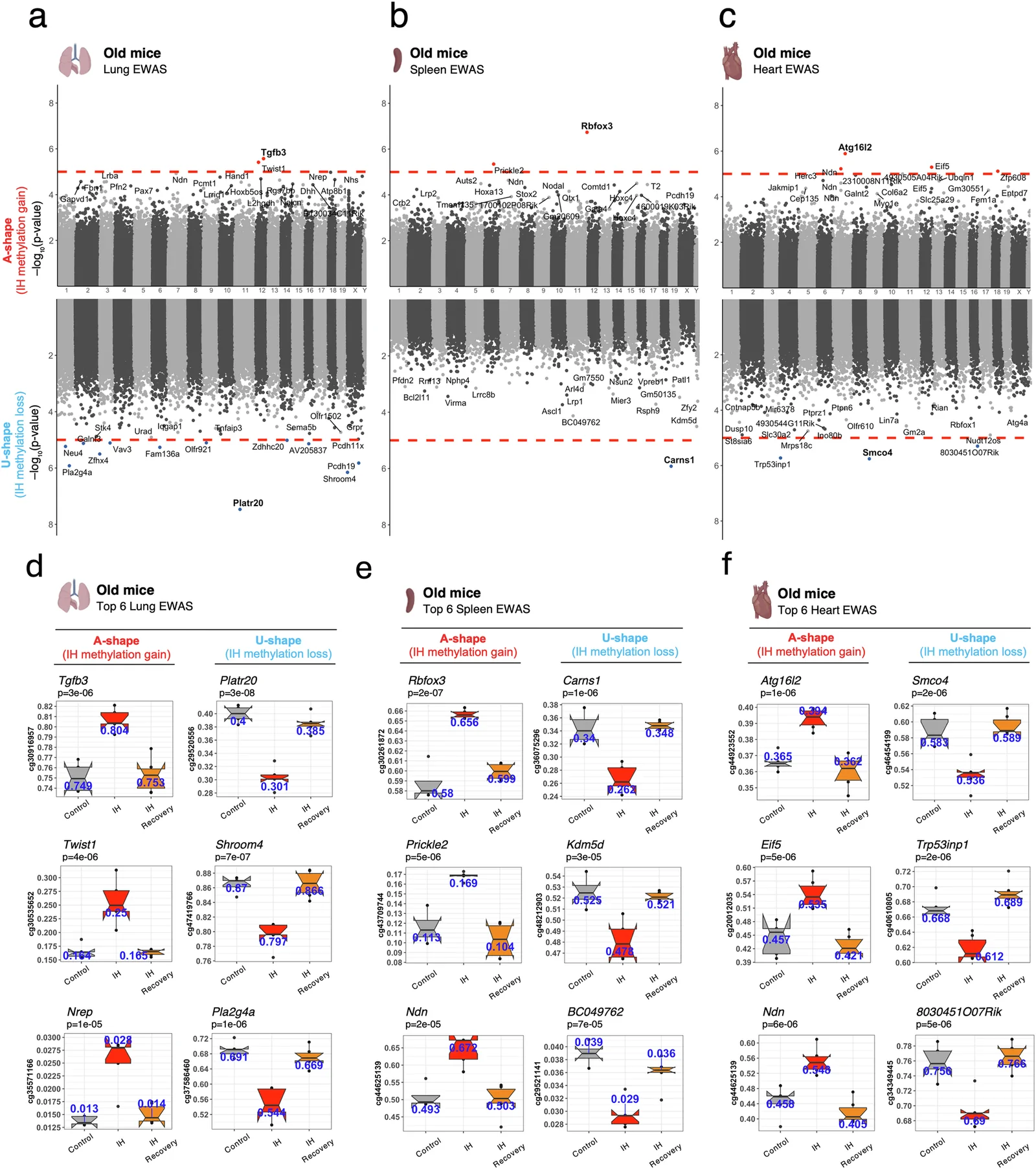
Old mice Epigenome-wide association studies (EWAS) in lung, spleen, and heart. **a**–**c** Miami plots for old **a** lung, **b** spleen, and **c** heart. The upper half depicts CpGs fitting a reversible A-shape model (hypermethylation during IH), and the lower half depicts CpGs that fit a reversible U-shape model (hypomethylation during IH). A red dashed line marks the threshold for significance (*p* < 1e⁻⁵); CpGs surpassing this threshold are highlighted in red (A-shape) or blue (U-shape). Y-axes represent statistical significance represented by the −log₁₀(*p* value). **d**–**f** Top differentially methylated CpGs exhibiting reversible hypoxia effects in old mice (“Italian Moka” plots, boxplots showing distribution trajectories) for **d** lung, **e** spleen, and **f** heart. Panels show the methylation levels at the top three CpGs per tissue. CpGs with positive (A-shape) effects are displayed in the left column; CpGs with negative (U-shape) effects are displayed in the right column. Y-axes represent methylation levels as well as the CpG sites across chromosomal position (mm10) for Control (dark gray), IH (red), and IH Recovery (orange).

Because it emerged that IH accelerated epigenetic aging in old but not in adult mice, we focused subsequent analyses on the old cohort (still providing results for adults in the supplemental figures and data). We therefore conducted tissue-specific Epigenome-Wide Association Studies (EWAS), comparing IH exposure against both baseline and recovery conditions. From these analyses, we identified CpG sites responsive to hypoxia as those showing significant, transient methylation changes during IH that were reversed upon recovery, exhibiting either an A-shaped trajectory for reversible methylation gain or a U-shaped for reversible methylation loss trajectory. To highlight the most robust reversible sites, we applied a conservative significance threshold of *p* < 1e⁻⁵ = 0.00001 (see Miami plot in Fig. 3a–c).

In old mice, IH was associated with two hypermethylated (*Tgfb3* and *Twist1*) and twelve hypomethylated CpG loci (top 10: *Platr20, Shroom4, Pla2g4a, Pcdh19, Zfhx4, Fam136a, Neu4, AV205837, Galnt3,* and *Vav3*) in the lung (Fig. 3a); two hypermethylated (*Rbfox3* and *Prickle2*) and one hypomethylated (*Carns1*) locus in the spleen (Fig. 3b), and four hypermethylated (*Atg16l2, Eif5, Ndn,* and *Zfp608*) and three hypomethylated (*Smco4, Trp53inp1,* and *8030451O07Rik*) loci in the heart (Fig. 3c). The list of the top 500 CpGs with hypermethylated and hypomethylated regions is provided for each organ (Supplementary Data 1–3).

Below, we briefly highlight the top three hyper- and hypomethylated genes identified in Fig. 3d–f, based on the CpG sites located closest to their respective genes. In the lungs, IH-induced hypermethylation was observed at CpG sites nearest to TGF-β3 (*p* = 3e^−6^), *Twist1* (*p* = 4e^−6^), and *Nrep* (*p* = 1e^−5^). The top hypomethylated sites were located near *Platr20* (*p* = 3e^−8^), *Shroom4* (*p* = 7.0e^−7^), and *Pla2g4a* (*p* = 1e^−6^) (Fig. 3d). Prior work indicates that hypoxia activates TGF-β3 signaling with context-dependent outcomes. While this pathway appears to protect cells from stress^29^ and particularly neurons from IH-induced oxidative stress^30^, in cardiopulmonary tissues TGF-β3 can conversely drive fibrosis and pathological remodeling^31,32^. This aligns with mechanisms of Endothelial-to-Mesenchymal Transition (EndMT), where Twist1 has been implicated as a therapeutic node in hypoxia-induced pulmonary hypertension^33,34^. Lastly, *Nrep* (also defined as P311) is transcriptionally upregulated by HIF1A and promotes glycolysis, proliferation, and metastasis in breast cancer^35^. Among hypomethylated loci, *Pla2g4a* falls within selective sweeps (arising when an advantageous genomic change rapidly increases frequency in a population, conferring fitness benefit) in high-altitude-adapted Tibetan pigs, suggesting broader relevance to hypoxic adaptation^36^. In the spleen, hypermethylated CpGs were detected proximal to the gene *Rbfox3* (*p* = 2e^−7^), *Prickle2* (*p* = 5e^−6^), and *Ndn* (*p* = 2e^−5^), while the top three hypomethylated sites were located proximal to Carns1 (*p* = 1e^−6^), *Kdm5d* (*p* = 3e^−5^), and *BC049762* (*p* = 7e^−5^) (Fig. 3e). *Rbfox3* is a neuronal splicing regulator that also controls the biogenesis of a subset of microRNAs^37,38^. *Prickle2* has recurrent associations with neurodevelopmental and neurological phenotypes^39,40^. *Ndn* (also referred as *Necdin*) physically interacts with ARNT2 and HIF1A, linking it to hypoxia-responsive transcriptional machinery^41^. Among hypomethylated loci, *Carns1* has been associated with tumor-suppressive/prognostic roles in breast cancer^42^, while *Kdm5d* demethylates p38α at K165 to restrain its activation and inhibit tumor progression^43^; ultimately, *BC049762*, associated with *Msantd5l*, lacks established oxygen-sensitive links and a clear functional role. In the heart (Fig. 3f), hypermethylation was observed near *Atg16l2* (*p* = 1e^−6^), *Eif5* (*p* = 5e^−6^), and *Ndn* (*p* = 6e^−6^), with hypomethylated CpGs found near *Smco4* (*p* = 2e^−6^), *Trp53inp1* (*p* = 2e^−6^), and the long non-coding RNA gene *8030451O07Rik* (*p* = 5e^−6^). *Atg16l2* is a modulator of autophagy with disease relevance^44^. Overexpression of *Eif5* (and its mimic 5MP) was found to perturb eIF2 function inducing ATF4-dependent stress-adaptive translation likely in the context of the integrated stress response (ISR)^45^. *Trp53inp1* has been shown to support neuronal homeostasis via autophagy/mitophagy pathways^46^. To further contextualize these findings, we quantified the magnitude of methylation change across baseline, IH, and recovery conditions and expanded the annotations for the CpG sites associated with reversible hypoxia effects. For each organ (lung, spleen, and heart), we report the genomic context of the top 20 hypermethylated (A‑shape) and top 20 hypomethylated (U‑shape) CpG sites, providing insight into their potential regulatory relevance. The methylation levels for these loci across all experimental conditions are presented in Supplementary Figs. 5–10. Although whole‑genome bisulfite sequencing (WGBS) data were not available in this study, we provide detailed regional mapping for these CpGs, including their genomic context (e.g., introns, exons, promoters) and their distances to the nearest transcriptional start sites (TSS), to better define the localized epigenetic response to intermittent hypoxia.

In adult mice, the analysis on lungs identified two hypermethylated (*Creg1 and Cryba4*) and 6 hypomethylated loci (*Srprb, Cacna2d1, Sox15, Mfsd13a, Ppm1f*, and *Ppp6c*) (Supplementary Fig. 11a). Among these, *Cacna2d1* was previously reported as a main hub gene closely related to hypoxia preconditioning^47^. *Ppm1f* has been implicated in the cardioprotective effects of exercise-derived circulating exosomes^48^. The spleen showed 33 hypermethylated loci (top 10 by significance: *Myo10, Tufm, Ptprm, Gm5640, Atp8a2, Catsperb, Gm6313, Cybrd1, Kazn*, and *Slc4a4*) and no hypomethylated loci (Supplementary Fig. 11b). *Myo10* is a potential target for early diagnosis and treatment of pulmonary hypertension under hypoxic conditions^49^. *Tufm* has been linked to hypoxia-related vascular remodeling, as its silencing inhibited the development of pulmonary hypertension by modulating mitochondrial autophagy via the AMPK/mTOR signaling pathway^50^. *Ptprm* was identified as prognostic for hypoxia- and mitochondria-related gene signatures predictive of lung adenocarcinoma^51^. *Slc4a4* expression was shown to be induced by hypoxia in triple-negative breast cancer, where regions of low oxygen promote acidosis and metastasis^52^. In the heart, we identified 3 hypermethylated (*Celf4, 9630013A20Rik, Foxj3*) and 1 hypomethylated (*Sptbn4*) loci (Supplementary Fig. 11c). *Foxj3* orchestrates a regulatory axis involved in pulmonary vascular acclimation to hypoxia^53^. As we did for the older cohort, we share the list of top 500 EWAS (Supplementary Data 4–6) and visualizations of the top 3 hypermethylated and top three hypomethylated CpG sites, along with their nearest annotated genes, for the lungs, spleen, and heart, respectively (Supplementary Fig. 11d–f). As with the older cohort, we also quantified the magnitude of methylation change and annotated the genomic context of the top reversible CpG sites in adult mice. For each organ, we provide the genomic locations of the top 20 hypermethylated (A‑shape) and top 20 hypomethylated (U‑shape) CpG sites, which may inform their potential regulatory impact. These analyses are presented in Supplementary Figs. 12–17.

To identify clusters of hyper- and hypomethylated CpG sites across three tissues and two ages, we examined those differentially methylated in at least three of the six possible groups (Supplementary Fig. 18a). Among these, the age-dependent sites hypermethylated only in the older group across all tissues (top 500 per tissue) included *Ndn, Tfap2a, Bcor, Bdnf, Eif5, HIF1A, Tmem164, Galnt2*, and *Gm73013*. Excluding the previously described *Ndn, Tfap2a* was found to act as HIF-1 co-player in hypoxia through SUMOylation-mediated regulation^54^. Hypoxia-driven epigenetic reprogramming alters the context-dependent role of *Bcor* in transcriptional regulation and cell fitness^55^. *Bdnf* has been consistently linked to VEGF and hypoxia signaling, including under diabetic and hypoxic conditions^56^, neurodevelopmental disorders and neuronal survival^57^, molecular and neuronal remodeling^58^, and sleep apnea^59^. HIF1A, as the canonical hypoxia-inducible factor, showed differential methylation across tissues. *Eif5* was also recently identified as an RNA switch transcript responsive to hypoxia in cell culture models^60^. The only consistently hypomethylated gene in the older group was Tnfaip8 (Supplementary Fig. 18b). *TIPE2*, encoded by *Tnfaip8l2*, inhibits hypoxia-induced Wnt/β-catenin pathway activation and EMT in glioma cells^61^.

### Hypoxia-reversible DNA methylation signatures are partially maintained in adult and old mice

To determine if the epigenetic response to intermittent hypoxia is conserved during aging, we compared the Z-statistics derived from the IH effect (specifically, IH vs. Baseline) in adult mice with those in old mice. We calculated Pearson correlations of these Z-scores for each of the three tissues to quantify the concordance of the IH response between the two age groups (Supplementary Fig. 19). In the lung, the hypoxia-induced methylation changes were negatively correlated between adult and old animals (*r* = −0.236; Supplementary Fig. 19a), which suggests a fundamental difference in the epigenetic response to hypoxia between these age groups. In the spleen, we observed a positive correlation (*r* = 0.391; Supplementary Fig. 19b), consistent with shared directionality across ages. In the heart, there was no correlation (*r* = 0.019; Supplementary Fig. 19c).

Below, we describe EWAS hits shared between the adult and old groups in response to hypoxia, along with annotations from the literature linked to oxygen modulation. In the lungs: hypermethylated *Eya2* lies downstream of an EGFR–HIF1A axis^62^; *Osm* (Oncostatin-M) augments HIF1A and drives both hypoxia-like metabolic reprogramming^63^ and macrophage hypoxia signaling^64^; *Il17f* reflects HIF-1–dependent Th17 programming since Il17f promoter shows H3/H4 hyper-acetylation in WT but not HIF1A−/−T cells^65^; hypomethylated genes included *Cd9*, a direct HIF1A transcriptional target under hypoxia^66^. In the spleen, *E2f3* links to hypoxia via either transcriptional activation of EPAS1/HIF-2α^67^ or hypoxia-induced mitophagy^68^. In the heart, hypermethylated *Hspa2* was found to modulate HIF-1–dependent programs in keratinocytes^69^, while *Col6a2* participates in hypoxia-modified collagen VI, which was observed to weaken lung endothelial barriers^70^; heart hypomethylated genes were *Nsmaf* (FAN) which aligns with early neutral sphingomyelinase activation during hypoxia/reoxygenation injury^71^, *Gpr174* deletion enhances post-ischemic (hypoxic) neovascular recovery^72^, and Cdh4 increases in the left ventricle under chronic sustained hypoxia^73^.

### Enrichment of CpG sites with reversible hypoxia-associated methylation changes

To explore the functional implications of the observed reversible methylation changes, we performed a genomic region–based enrichment analysis using the GREAT software tool (see Methods for more details). For this analysis, we selected the top 500-CpG sites with positive (A-shaped pattern) and the top 500 with negative (U-shaped pattern) associations from our tissue-specific EWAS in old mice and adult mice, respectively. A complete summary (for old mice) is provided in Supplementary Data 7–9.

**Fig. 4:**
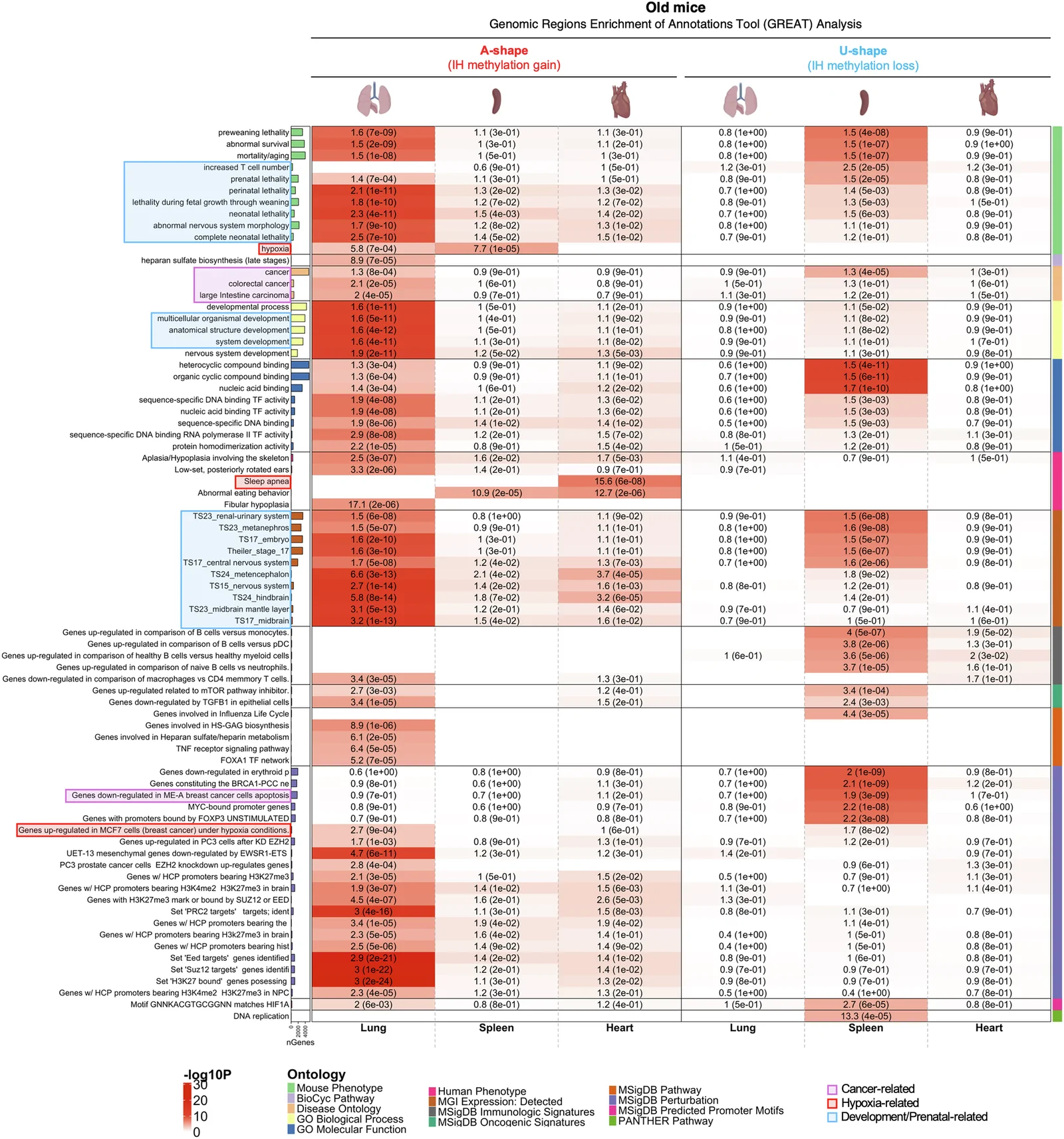
Functional enrichment (GREAT analysis) of reversible CpGs in old mice highlights developmental, hypoxia/aging, and cancer pathways. GREAT enrichment analysis of the top 500 CpGs with positive and negative associations is structured into six panels: three for reversible methylation gain (A-shape) and three for reversible methylation loss (U-shape), each subdivided by tissue, respectively lung, spleen and heart. The bar plots in the first column display the total number of genes within each annotation set, adjusted for background. Each cell in the main panels reports both the fold change and the corresponding unadjusted hypergeometric *p* value. The color gradient of each cell corresponds to the −log10 (*p*). A column color band on the right denotes the ontology source of each gene set. The pathways are highlighted into three macro-categories: hypoxia/aging-related (red labels), developmental/prenatal-related (blue labels), and cancer-related (purple labels).

As summarized in Fig. 4, in old mice, the EWAS of reversible IH effects showed the strongest enrichment in the lung, particularly in pathways associated with methylation gains. A recurring subset of pathways, identified across multiple ontologies, was found to be associated with prenatal, neonatal, and postnatal development. This is notable given a recent study reporting that accelerated epigenetic aging in newborns with congenital heart disease (CHD) was strongly associated with reduced blood oxygen saturation^74^. Notably, all three significantly enriched pathways within the Disease Ontology dataset were cancer-related (the association between hypoxia and cancer is well established). Another major class of enriched pathways was linked to hypoxia and aging across multiple ontology resources. This convergence highlights the broad relevance of hypoxia-related epigenetic regulation, spanning developmental, oncogenic, and aging processes. Furthermore, the CpGs with a positive association in the lung EWAS of old mice exhibited enrichments in hypoxia-related pathways within the Mouse Phenotype ontology (*p* = 7e^−4^; fold change FC = 5.8) and an HIF1A -related motif in the MSigDB Predicted Promoter Motifs ontology (*p* = 6e^−3^; FC = 2.3).

The significant enrichments in old mice were not replicated in adult mice (Supplementary Fig. 20, with a complete summary in Supplementary Data 10–12 for adult mice). Overall, our findings suggest that the association between these CpGs and hypoxia-related pathways is an age-specific phenomenon, rather than a general effect of reversible hypoxia across the lifespan.

### DNA methylation remodeling under IH mainly targets the chromatin states implicated in PRC2 binding regions

We then performed a chromatin state analysis to annotate the CpGs exhibiting reversible hypoxia effects in old mice. The analysis included the top 500 CpGs with positive and negative associations from the EWAS (Supplementary Fig. 21 and Supplementary Data 13–18).

Universal ChromHMM annotation shows a striking enrichment of methylation gain (A-shape) CpGs at bivalent promoters 1 (BivProm1: *p* = 3.4e⁻⁴⁹; FC = 6.3) and 2 (BivProm2: *p* = 1.7e⁻⁷; FC = 2.7) and Polycomb repressive complex 2 (PRC2: *p* = 5.7e⁻¹³; FC = 4), followed by Polycomb Repressive 1 (ReprPC1: *p* = 9.9e⁻³; FC = 2.1).

In contrast, methylation loss (U-shape) CpGs were depleted in these states (FC <1). This pattern parallels pan-mammalian aging EWAS (Supplementary Fig. 22) and suggests that IH-responsive CpGs in old mice reside in developmentally poised, Polycomb-regulated chromatin. Notably, previously reported GREAT analysis corroborates these chromatin state results^10^. Specifically, several PRC2-related functional sets (including H3K27me3) were robustly enriched among CpGs that gain methylation with age in the pan-mammalian EWAS—see Fig. 7 in ref. ^10^. While the enrichments were weaker, similar patterns of enrichment in BivProm, ReprPC1, and PRC2 were also observed in the spleen and heart EWAS. These findings, however, were specific to the old mice and were not replicated in the adult mice (Supplementary Fig. 23 and Supplementary Data 19–24).

Next, in the lungs of old mice exposed to intermittent hypoxia, we observed increased average percent methylation levels at PRC2 binding sites compared to a combined group from controls and IH recovery (*p* = 4.9e^−3^), controls alone (*p* = 0.016) and IH recovery alone (*p* = 0.042, Supplementary Fig. 24). This indicates that PRC2 methylation tracks reversible hypoxia with an A-shape. Similar reversible PRC2 mean methylation changes were also observed in old spleen and in old heart with borderline significance (*p* = 0.06 and *p* = 0.078, respectively) and were only weakly replicated in the spleen of the adult mice (*p* = 0.061).

While the hg19-based ChromHMM assembly enabled comparisons with pan-mammalian aging EWAS, we also performed a chromatin state annotation specifically for the mouse genome using the 100-state full-stack model (Methods). We observed that the top two chromatin states overlapping with PRC2 binding regions remain consistent across both models. In the mouse-specific annotation, mBivProm2 showed the highest overlap (containing 29% of the array’s CpGs), followed by mBivProm1 (17%). This mirrors our findings in the human ChromHMM model, where BivProm1 (21%) and BivProm2 (20%) were likewise the primary states associated with Polycomb-repressed regions.

The functional patterns remained comparable between the two annotations, further supporting the robustness of our human-based results. For the top CpGs positively associated with the old lung EWAS, enrichment in bivalent promoter regions was consistent across models (FC = 5.1, *p* = 7.5 × 10^−41^ in the mouse model). Notably, the enrichment in the transcription start site (TSS) for this same old lung EWAS, which was modest in the human model (TSS1: FC = 1.8, *P* = 2.2 × 10^−3^), was confirmed here and appeared even more distinct when using the mouse-specific mTSS1 annotation (FC = 7.1, *p* = 4.1 × 10^−79^; Supplementary Fig. 25 and Supplementary Data 25–30 for the three organs, each with sheets for positive and negative associations). The results for adult mice, not further discussed here, are provided as Supplementary Fig. 26 and Supplementary Data 31–-36).

### Hypoxia-associated motif enrichments detected using HOMER analysis

To elucidate regulatory mechanisms underlying age-related changes in DNA methylation, we performed motif enrichment analysis using HOMER (Hypergeometric Optimization of Motif EnRichment), which identifies transcription factor binding motifs statistically overrepresented proximal to differentially methylated CpG sites (a region size of 200 bp) compared to the background genome based on our 320k array. This approach provides insight into upstream regulators that may contribute to epigenetic remodeling across the lifespan and has previously been applied in aging and epigenetic clock studies to implicate specific transcription factors in age-associated methylation dynamics^6^.

A total of 21 de novo motifs were identified in old mice tissues with an enrichment of *p* < 1e^-12^ (Supplementary Data 37–42). Specifically, for the CpGs with a positive association in the lung EWAS, 12 motifs were identified, including the E-box(bHLH) motif, a composite motif representing the CEBP:AP1(bZIP) heterodimer, and motifs for NFATC4, among others. The involvement of these transcription factors (TFs) in the hypoxic response has been well-established in the literature. For instance, Hypoxia-inducible factor 1-alpha is a key regulator that directly binds to the E-box motif^75^. Additionally, a member of the CEBP family, CEBPD, has been identified as a master transcriptional factor for hypoxia, augmenting hypoxia-induced invasion through the EGFR/PI3K pathway^76^. An earlier study also demonstrated that hypoxia activates several genes known to be inducible by AP1 in endothelial cells^77^. The calcineurin-NFATc4 signaling pathway is a major regulator of neural stem cell biology under hypoxic conditions^78^, further supporting its role in the hypoxic response. In contrast, no de novo motifs were significantly enriched for CpGs with a negative association in the lungs. A few de novo zinc finger transcription factors were identified for the heart and spleen EWAS.

Enrichments for known motifs were identified, too, though they were generally of modest statistical significance (Supplementary Data 43–48). For instance, the CEBP homodimer motif was found to be associated with a positive enrichment in the lung EWAS, with a marginal unadjusted *p* = 0.01 and FDR = 0.36.

Only a few de novo motifs were enriched with the adult mice EWAS, and none of them replicate the motifs associated with the old mice EWAS (Supplementary Data 49–54). On the other hand, known motifs were markedly more abundant in the adult mice EWAS, with a total of 56 distinct transcription factors identified (FDR <0.1, Supplementary Data 55–60). Notably, 21 of these belonged to the ETS family. Among the ETS family, seven transcription factors were enriched in both age groups, suggesting a shared regulatory mechanism. For example, ETS1 was identified with a negative association in the adult spleen (*p* = 1e^−12^), old spleen (*p* = 1e^−6^), and old heart (*p* = 1e^−3^), as well as with a positive association in the adult lung (*p* = 1e^−3^; Supplementary Fig. 27). These results are aligned with cooperative HIF-2α/ETS1 regulation at composite elements controlling endothelial targets such as Flk-1/Kdr^79^ and with tumor-context data implicating ETS1 in hypoxia-inducible gene programs^80^.

Finally, with positively associated spleen sites, Pbx2 emerged as the top motif; Pbx factors have been shown to drive perinatal pulmonary vascular adaptation (an exquisitely oxygen-sensitive process), supporting plausible PBX involvement in hypoxia-responsive regulation^81^. In terms of the heart, NF1 was most enriched, whereas in the lung, ZNF768 showed the strongest enrichment.

### Hypobaric hypoxia elevates epigenetic age acceleration during human acclimation

To further explore the hypothesis that fluctuations in oxygen level drive epigenetic aging, we analyzed publicly available data of DNA methylation change in humans during acclimation from sea level to a high-altitude hypobaric hypoxia environment. We obtained blood DNA methylation data from 19 young participants (range: 19–23 years), from the AltitudeOmics project^82^. These participants acclimatized to high altitude (ALT) at 5260 meters (m) from near sea level over 16 days. Each subject was initially studied at baseline near sea level, followed by measurements at 5260 m at noon on day 1 and at 1 PM on days 1, 7, and 16. A total of 35 epigenetic clocks and DNA methylation-based biomarkers were estimated at each of the five time points, including the Horvath pan-tissue clock, DNAmGrimAge2, DunedinPACE, and both Universal Clocks for assessing the pace of epigenetic aging during acclimatization (see Methods for more details).

**Fig. 5:**
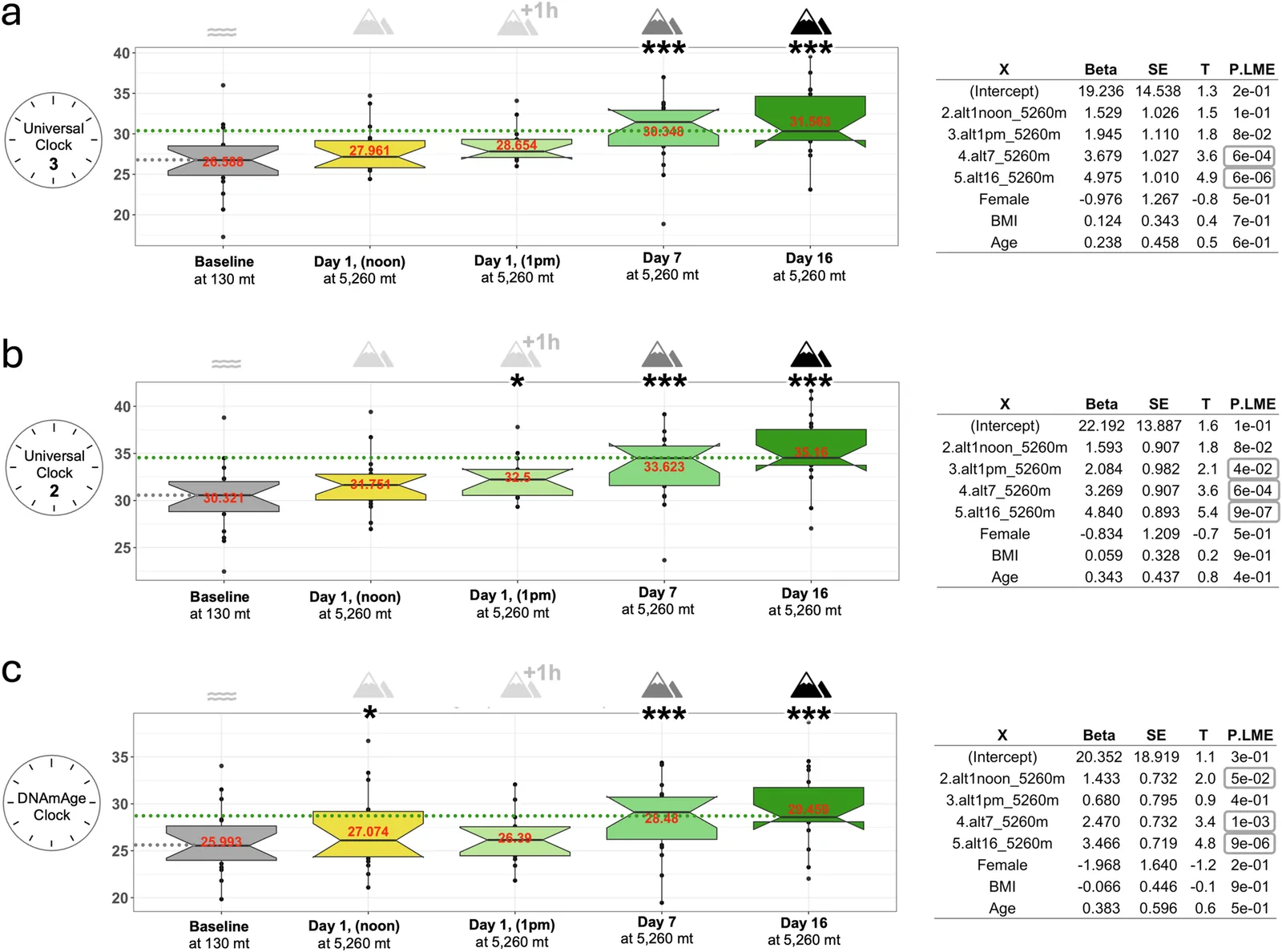
Epigenetic clocks track human acclimatization to hypobaric hypoxia. **a–c** Longitudinal changes in DNA methylation age (DNAmAge) across five time points for **a** DNAmGrimAge2, **b** UniversalClock2, and **c** UniversalClock3. Y-axes represent estimated DNAmAge (years). X-axes represent the timeline including baseline (130 m) followed by high-altitude exposure (5260 m) at Day 1 (Noon and 1 PM), Day 7, and Day 16. Mean DNAmAge estimates for each time point are reported in red; the range of estimates is displayed in the panel titles. Accompanied tables display results from linear mixed-effects (LME) model analysis associating DNAmAge with time point (relative to baseline), adjusted for gender, BMI, and chronological age. The *P* values reported from LME analysis are unadjusted. The asterisks represent statistical significance (*P.LME ≤ 0.05; **= P.LME ≤ 0.01; ***= P.LME ≤ 0.001).

In spite of the substantial difference in hypoxia exposure, the results of this analysis are consistent with the findings in the mouse experiment by showing that DNA methylation age significantly accelerates in humans over acclimatization to hypobaric hypoxia; this relationship remains robust in linear mixed-effects (LME) models even after adjusting for gender, BMI, and chronological age (Fig. 5, Supplementary Fig. 28, and Supplementary Data 61). After rapid ascent to 5260 m, the participants showed a significant increase in DNAmGrimAge2 of 1.43 years on day 1 noon time (unadjusted *p* = 5.0e-02, the table accompanying Fig. 5a). DNAmGrimAge2 continued to increase, peaking after 16 days at 5260 m with an increase of 3.47 years (*p* = 9.0e-06). Consistent with the GrimAge2 results, Universal Clocks 2 and 3 showed similar significant increases (Fig. 5b, c). Notably, all three clocks showed the highest DNAmAge estimates on day 16, with increases ranging from 3.47 to 4.84 years (*p* ≤ 9.0e-07). As listed in Supplementary Data 61, a variety of other epigenetic clocks echoed these findings on day 16, including the Principal Component version of Horvath’s pan-tissue DNAmAge (PCHorvath1), with a Beta of 3.702 years (*p* = 7.0e-05), DunedinPACE (β = 0.086, *p* = 2.0e-05), and the Centenarian clock (ENCen40 + ) (β = 2.635 years and *p* = 9.0e-06).

Furthermore, several DNAm-based estimators of plasma proteins, the components of GrimAge2, also tracked acclimatization with significant elevation on Day 16 (*p* < 0.001). These included DNAm estimates of Adrenomedullin (ADM, *p* = 2.0e-06), Beta2-microglobulin (B2M, *p* = 1.0e-05), Growth Differentiation Factor 15 (GDF15, *p* = 1.0e-04), Cystatin-C (*p* = 6.6e-06), Tissue inhibitor metalloproteinases 1 (TIMP1, *p* = 4.0e-04), and log-transformed C-reactive protein (CRP, *p* = 7.0e-04). These robust findings were replicated in their Principal Components versions, such as PC-B2M (*p* = 3.0e-07).

We also examined changes in mean PRC2 methylation and observed only a modest increase of 0.007 at Day 16 (*p* = 2.0e-02; Supplementary Fig. 29 and Supplementary Data 61), based on the same linear mixed-effects model adjusted for age, sex, and BMI.

We next performed EWAS using the same LME framework, with the five consecutive time points encoded as a continuous variable in days (Methods). This analysis identified many CpGs whose methylation levels were associated with duration at high altitude, including 597 CpGs surpassing *p* < 1.0e-07 (Supplementary Fig. 30). Of these, 528 CpGs gained methylation with increasing duration, including cg20306694 in *BIN2* (*p* = 3.5e-14), cg16782524 in *AGO2* (*p* = 2.5e-13), and cg14576319 in *TNIK* (*p* = 4.8e-13), whereas 69 CpGs lost methylation, including cg06934654 in *ACSF3* (*p* = 1.6e-11), cg00900735 in *GRB2* (*p* = 3.8e-10), and cg19865472 in HCN2 (*p* = 8.1e-10).

Among the top 10,000 CpGs showing increased methylation with increasing duration at high altitude (*p* < 5.6e-04), we observed enrichment in PRC2 binding regions, although the effect size was only modest (FC = 1.4, *p* = 2.3e-11; Supplementary Fig. 31). In contrast, the top 10,000 CpGs showing decreased methylation were strongly depleted in PRC2 binding regions (FC = 0.1, *p* = 3.9e-84). A similar bidirectional pattern was also observed for bivalent promoter states, particularly BivProm1 and BivProm2 (Supplementary Fig. 31). Although several active enhancer states showed stronger enrichment signals, they did not display the same mirror pattern. Despite the human data being generated from blood in young individuals, these PRC2-related patterns modestly parallel our findings from lung EWAS in the aged group.

## Discussion

In this study, we show that intermittent hypoxia induces a robust, age-dependent acceleration of epigenetic age in vivo. This effect was restricted to old mice, suggesting that the old epigenome possesses a unique vulnerability to hypoxic stress. It is also possible that intermittent hypoxia acts as a greater stimulus in old mice because their tissues are already hypoxic to start with, and their capacity to adapt to hypoxia is impaired. Although the recovery period examined here was limited, it is possible that old tissues may exhibit slower but potentially more complete reversal of IH-associated epigenetic age acceleration over longer intervals. Determining the full kinetics of recovery will require extended longitudinal studies. Of note, the oscillatory intervention elicited biological responses distinct from constant hypoxia, as animals underwent repeated nadir–peak oxygen transitions at a frequency (one cycle every 2:30 min) known to activate transcriptional and oxidative pathways specific to intermittent hypoxia rather than to a static average oxygen level.

We posit that epigenetic clocks partially track the failure of physiological homeostatic mechanisms aimed at maintaining oxygen delivery. In adult mice, robust compensatory systems (such as flexible metabolic strategies^83^ and retained vascular capacity^84^) may effectively buffer hypoxic stress, resulting in no epigenetic clock advancement. Conversely, in older animals, these compensatory systems fail. The epigenetic age acceleration observed, therefore, reflects a secondary molecular recording of this systemic failure, occurring in a landscape where chromatin is already compromised, and possibly a certain degree of hypoxia is already present at baseline. Epigenetic clocks are indicators of biological age, but they are not the same as biological age itself. We acknowledge that hypoxia signaling may trigger a molecular response that is captured by the epigenetic clocks, which suggests, but not necessarily prove that it affects the pace of biological aging. However, several features of our experimental design are consistent with causation. First, IH was applied as a controlled intervention before measuring epigenetic age, establishing temporal precedence; the age acceleration was not present at baseline and emerged only after IH exposure. Second, the magnitude of acceleration correlated with tissue-specific oxygen exposure (lungs > spleen > heart), which would be unexpected if the signal merely reflected a generic stress response unrelated to oxygen biology. Third, the substantial reversal of epigenetic age upon return to normoxia argues against a permanent confounding and is consistent with a direct, modulable effect of oxygen availability on the methylation landscape. Nonetheless, orthogonal experiments that establish that hypoxia affects other aging markers, such as phenotypes, tissue-level functional assays or proteomic aging signatures, would be required to further support the hypothesis of causality. Of note, this experiment was designed based on an “a priori” hypothesis, that was fully confirmed by the data.

To assess translational relevance, we analyzed human blood data from the AltitudeOmics project^82^. In contrast to the intermittent nature of our mouse model, these young individuals (19–23 years) underwent short-term but chronic hypobaric hypoxia. The fact that both intermittent and sustained hypoxia accelerate aging strengthens the argument that oxygen fluctuation itself is the driver. The kinetics of the human response were remarkably fast, with acceleration observed immediately upon ascent and peaking on Day 16. This finding was robustly replicated across a diverse range of epigenetic clocks (including DNAmAge, DNAmGrimAge2, and DNAmTIMP1). Recent population data confirm that these effects persist beyond the acute phase. In a large-scale study of Han Chinese, Cheng et al. compared native lowlanders (*n* = 462) to acclimatized newcomers (*n* = 299) residing on the Tibetan Plateau for a median of 3 years^85^. Despite the acclimatization, the highland group exhibited significantly faster epigenetic aging compared to their lowland counterparts. These combined datasets reinforce the concept that whenever homeostatic limits are exceeded (whether due to age-related decline in mice, extreme environmental challenge in young humans, or chronic high-altitude residence), epigenetic age advances. Unlike observational studies, often confounded by lifestyle factors, our controlled design isolates hypoxia as the unique exposure driver.

Whether hypoxia induces similar shifts at hemi‑methylated or imprinted CpG sites (see dedicated paragraph in the Discussion) in humans remains largely unexplored. Studies of obstructive sleep apnea (OSA), a clinical condition characterized by chronic intermittent hypoxia, have reported widespread DNA methylation changes in blood and airway tissues, including CpG‑specific alterations in hypoxia‑responsive genes such as *TLR2* and *TLR6*^86^. A recent pooled analysis of human OSA cohorts likewise identified hundreds of differentially methylated genes and demonstrated that intermittent hypoxia remodels immune, metabolic, and oxidative‑stress pathways at the epigenetic level^87^. Additional mechanistic work in rodent models shows that long‑term intermittent hypoxia can induce persistent, methylation‑dependent repression of antioxidant genes, linking hypoxia exposure to stable epigenetic remodeling^88^. Despite these findings, most available human datasets lack allele‑specific resolution, limiting the ability to determine whether hypoxia‑responsive CpGs overlap with known hemi‑methylated or imprinted loci. A recently published atlas of allele‑specific DNA methylation across human tissues provides a valuable reference for future work in this direction^89^. Our analysis of the AltitudeOmics dataset did not allow such an assessment because the Illumina 450 K array lacks the resolution needed for allele‑specific methylation. These gaps highlight the need for future human studies using whole‑genome bisulfite sequencing or long‑read approaches capable of resolving allele‑specific methylation states.

Beyond these specific findings, a broader link between oxygen modulation and aging is emerging. An analysis of 51 human intervention studies found that hyperbaric oxygen therapy (HBOT) reduced many epigenetic clocks^90^, supporting the view that oxygen availability functions as a tunable lever reversing epigenetic age. Skin-omics work implicates hypoxia signaling in accelerated aging^91^, and reviews identify hypoxia-inducible factors as key regulators of aging-associated transcriptional programs^92^. Conversely, increasing oxygen availability appears to reverse these trends. However, the relationship between oxygen and aging is not purely linear. Counter to our findings, an in vitro study suggested that lowering oxygen tension delayed DNA methylation aging in human fibroblasts^93^, and Rogers et al. recently reported that chronic hypoxia extended lifespan in a progeroid mouse model^94,95^. We speculate that these apparent contradictions reflect a hormetic dose–response relationship: while the chronic, mild stress in the progeroid model may trigger adaptive repair pathways, the intermittent stress in our aged model likely overwhelms homeostatic capacity, driving epigenetic aging.

Among the tissues examined, the lungs exhibited the strongest epigenetic clock acceleration and the most significant enrichment of reversible CpG sites. Biological themes consistently emerging from these reversible sites included developmental programs and bivalently regulatory regions: genomic loci marked by both repressive H3K27me3 (written by PRC2) and activating H3K4me3 in stem cells. The PRC2 target sites are known to gain methylation with age across all mammalian tissues^4,10,96,97^. To understand the enrichment for H3K27me3, one must consider the dual role of oxygen sensors in chromatin regulation. The H3K27 demethylase KDM6A (UTX) functions as a direct oxygen sensor; its inactivation during hypoxia prevents demethylation, theoretically locking cells in a stalled, repressive chromatin state^98^. This aligns with findings that acute hypoxia increases H3K27me3 at bivalent promoters via KDM6A inactivation^99^. However, reconciling this acute accumulation of H3K27me3 with our finding of DNA hypermethylation (which usually antagonizes H3K27me3) requires a nuanced view of PRC2 dynamics under stress. Recent evidence indicates that hypoxia does not merely enhance global repression but fundamentally reprograms PRC2 targeting. Under hypoxic conditions, PRC2 is often co-opted to drive specific malignant programs rather than maintaining homeostatic bivalency^100^. This hijacking model is substantiated by findings in breast cancer, where hypoxia dampens canonical SUZ12/EED-dependent repression while redirecting EZH2 to partner with FoxM1^101^. Similarly, hypoxia-induced recruitment of EZH2 has been shown to silence specific regulators in immune and tumor cells^102,103^. We propose that intermittent hypoxia creates a tug-of-war for PRC2 occupancy. While PRC2 is hijacked by hypoxic signaling pathways (HIF1A–EZH2 crosstalk^104,105^) or redistributed to non-canonical targets, the protective shield over bivalent regulatory regions and PRC2 target sites is temporarily eroded. It is likely this transient loss of continuous PRC2 occupancy at poised promoters that provides the mechanistic window for DNA methyltransferases to act, bridging the gap to age-related hypermethylation^10,97^. The interpretations regarding the PRC2 role will require targeted experimental validation.

A subset of CpG sites exhibiting reversible methylation dynamics mapped to genes with known monoallelic regulatory features. Among these, Ndn (Necdin) is the only classically imprinted gene; it is maternally imprinted and paternally expressed and represents a well-established example of parent-of-origin–dependent monoallelic expression^106^. Its presence among the top hypermethylated CpGs in old mice is consistent with the idea that loci with a preexisting hemi-methylated state may be particularly sensitive to additional methylation changes under hypoxic stress. Two additional genes, Kdm5d and Pcdh19, also exhibit monoallelic expression, although through mechanisms unrelated to imprinting. Kdm5d is Y-linked and therefore inherently monoallelic in males, whereas Pcdh19 is X-linked and subject to random X-inactivation, resulting in mosaic monoallelic expression in females^107^. Importantly, the remaining genes associated with CpG sites in Fig. 3d–f are not known to be imprinted or monoallelically expressed based on current imprinting catalogs. These observations suggest that imprinting or allele-specific regulation is unlikely to account for the majority of the reversible methylation patterns we observed. Instead, the intermediate methylation levels detected in bulk tissue are more plausibly explained by cellular heterogeneity (namely, the coexistence of fully methylated and unmethylated states across different cell types) rather than true allele-specific methylation within individual cells. This interpretation is particularly relevant for complex tissues such as lung and spleen

Our study has limitations. First, the sample size was relatively small (total *n* = 35 after quality check); future work should include male mice and multiple strains to assess generalizability. A further limitation is that bulk-tissue DNA methylation profiling cannot distinguish between cell-intrinsic methylation changes and shifts in cell-type composition. While reference-based deconvolution is not currently feasible for the mammalian methylation array in mouse tissues, the reversible design of our EWAS (selecting CpGs that change under IH and revert during recovery) partially mitigates this concern, as permanent cell-type shifts would be less likely to produce such reversible patterns. Additionally, the design featured a fixed 1-month exposure and 1-month recovery; varying these durations would clarify dose–response relationships and the limits of reversibility. We profiled only three mouse tissues; expanding to a multi-organ panel is necessary to quantify tissue-specific variability in IH-associated aging, and as well a longitudinal sampling design would better resolve individual aging trajectories. Furthermore, our translational analysis was constrained by the demographics and design of available human datasets. The AltitudeOmics cohort consists exclusively of young adults, which precludes a direct comparison to the age-dependent vulnerability observed in our older mice. While we observed significant acceleration even in this young group, we speculate that hypoxic stress would elicit a more pronounced epigenetic response in elderly subjects. Additionally, the human data lacks post-exposure longitudinal tracking, leaving it unclear whether the observed epigenetic age acceleration in humans is as reversible as that seen in our murine model. Future human studies should prioritize the inclusion of older cohorts and longitudinal recovery sampling, as well as targeted analyses of DNA methylation changes within gene regulatory regions to better parallel the mechanistic insights gained from animal models. IH may influence chromatin‑associated pathways, but the present data do not allow mechanistic inference; integrating proteomic and splicing readouts is essential to delineate how intermittent hypoxia reshapes downstream regulation beyond DNA methylation. Lastly, RNA‑seq data have not yet been generated as part of the SIHAM study; our experimental design focused on DNA methylation profiling using the mammalian methylation array. Paired RNA‑seq and ideally allele‑resolved using F1 hybrid crosses or long‑read RNA sequencing would be valuable for determining whether the observed methylation dynamics correspond to changes in allele‑specific gene expression. We consider this a current limitation and a priority for future work, as such experiments would help distinguish true allele switching from shifts in cell‑type composition as explanations for the observed methylation trajectories.

A growing body of evidence shows that stress can transiently accelerate epigenetic age, with effects often proving reversible upon stress removal^108^. Our in vivo findings support a bidirectional, reversible model in which hypoxia accelerates epigenetic aging in older mice. Mechanistically, we situate these changes within a framework of compromised PRC2 dynamics and canonical hypoxia-responsive networks. This study demonstrates that hypoxia remodels the methylome in both mice and humans, yet allows for a substantial rebound after reoxygenation. We propose oxygen availability as a tunable lever to potentially restore chromatin health and reverse epigenetic aging trajectories.

## Methods

### Animal model and intermittent hypoxia intervention

This study was performed at the National Institute on Aging (NIA, NIH), as part of the Study of Intermittent Hypoxia in Aging Mice (SIHAM). The experimental cohort consisted of 36 female C57BL/6 mice, allocated to two age groups: adult mice (~11 months) and old mice (~23 months) for a total of *n* = 18 per age group (*n* = 6 per group for all age and exposure conditions).

Exposure to intermittent hypoxia (IH) was achieved using the OxyCycler A84XOV system (Biospherix). Cages were placed in a controlled 82-liter capacity chamber. To maximize the chamber capacity, we group-housed up to four mice per cage. Since males might display territorial aggression and stress-induced fighting episodes, in this study, we used female mice only, as a reflection of practical housing constraints.

Gas inflow was delivered into a sealed chamber equipped with internal circulation fans, ensuring rapid homogenization of the airflow. Oxygen consumption by the mice represents a negligible fraction of total chamber volume and does not measurably alter the oscillation profile. Oxygen levels were then precisely controlled at predetermined times based on continuous feedback from an internal oxygen sensor. The oscillation parameters utilized in our study (21 to 5% O₂, 2:30 min/cycle with 24 cycles/h for 2 h daily gradual acclimation followed by 6 h/day for 1 month) were specifically selected to align with the severe IH ranges established in the literature. For example, published chronic IH models utilize oxygen nadirs ranging from 4.5 to 10%, with total exposure times spanning from 21 days up to 28 weeks^109–112^. The system was optimized by minimizing noise from gas release, and lighting, temperature, and humidity were maintained at stable levels throughout the experiment.

To verify the programmed oscillatory oxygen profile, we performed validation tests in the same chamber without mice (data not shown). Three empty cages were placed inside the chamber. Oxygen levels were recorded simultaneously in two cages and at the outflow using PreSens Microx4 fiber-optic sensors and an OxiGraf analyzer. These measurements showed minimal spatial variation across cages, confirming that the internal circulation fans homogenize the chamber atmosphere and that the oscillatory oxygen pattern is uniform throughout.

The protocol started with a 5-day acclimation period, during which the daily 8-h hypoxic exposure gradually intensified, with oxygen levels cycled down from an initial 21–15% range to a final 21–5% range. Following the preparatory experimental acclimation, IH intervention included a daily 2-h gradual transition phase when oxygen levels were cycled from an intermediate 21–15% range, gradually moving towards the full 21–5% O₂ range. This was then followed by a 6-h period of consistent cycling between 21 and 5% O₂. Each complete cycle lasted 2 min and 30 s (2:30 min), comprising the hypoxia and normoxia plateaus plus the transition ramp times. This approach minimized hypoxic shock and enabled both adult and aged mice to transition safely to the long-term IH protocol. We randomly allocated mice to three experimental conditions: untreated (unt, negative controls), 1 month of IH, or 1 month of IH followed by 1 month of normoxia recovery (IH recovery, IHrec).

Tissue collection was performed in cohorts of 12 mice for each age group during the last 3 days of the experimental period, within a 4-h window between 8 a.m. and 12 p.m. Mice were euthanized using a two‑stage procedure consisting of inhalation anesthesia followed by exsanguination and decapitation. Animals were first anesthetized with 2–5% inhaled isoflurane in oxygen until a surgical plane of anesthesia was achieved. While animals remained under deep anesthesia, transcardial perfusion was performed by inserting a needle into the left ventricle and infusing ~20 ml of phosphate‑buffered saline, with an incision in the right atrium to allow outflow. Perfusion was conducted to remove circulating blood from the organs, as residual blood could have confounded downstream analyses. Exsanguination was confirmed visually by blanching and color change of tissues. Following completion of perfusion, death was ensured by decapitation, after which organs were dissected for analysis. This approach minimized animal suffering while enabling the collection of blood‑depleted tissues. Lung, spleen and heart tissues were harvested from humanely euthanized mice, snap-frozen in liquid nitrogen, and stored in freezers at −80 °C until processing for DNA extraction and hypoxia-inducible factor 1-alpha (HIF1A) quantification. All animal procedures and housing adhered to the guidelines of the National Research Council’s Guide for the Care and Use of Laboratory Animals and were approved by the Animal Care and Use Committee of the NIA Intramural Research Program (IRP). Animals were maintained on a standard diet, housed on a 12:12 light:dark cycle, and kept at a controlled temperature of 20–24 °C.

### HIF1A detection using Western blot analysis and ELISA

To determine expression levels of HIF1A in lung tissue (from 22 mice in total), 20–40 mg specimens were sectioned on a dry-ice-chilled glass plate. Each sample was placed into a 2-mL tube containing tissue homogenizing beads (Precellys, Bertin Technologies) and 800 µL RIPA buffer supplemented with protease inhibitors (Halt Protease and Phosphatase Inhibitor Cocktail, Thermo Fisher Scientific). Samples were homogenized in a pre-cooled homogenizer (Precellys 24, Bertin Technologies) for 15 s, and then centrifuged at 4 °C for 5 min (12,000×*g*). The obtained lysates were aliquoted (30–50 µL reserved for the BCA assay, Thermo Fisher Scientific, #23225) and protein concentrations were adjusted to 2 µg/µL. Then, 30–40 µg of protein per lane was separated by Western blot. Membranes were probed with HIF1A antibody (1:1000, Abcam) overnight at 4 °C, followed by incubation with an anti-rabbit secondary antibody (1:1000, in 5% milk/TBST) for 1 h at room temperature. Detection was performed using undiluted ECL reagent, with HIF1A verified at ~100 kDa using a colorimetric marker. Western blot quantification was performed using two distinct analytical approaches based on the experimental design. For all analyses, raw band intensities were first normalized to actin. For baseline comparisons, adult and old samples were evaluated on the same gel. For the two-month intervention study, adult and old samples were run on separate gels; to account for this, the actin-normalized values were subsequently expressed as a fold change relative to the mean of the age-matched untreated (Unt) samples. Baseline differences in protein expression between adult and old cohorts were evaluated using an unpaired Student’s *t*-test. For the two-month intervention study, differences were evaluated using a two-way ANOVA to determine the main effects of the intervention (Unt, IH, IH Recovery) and age, as well as their interaction. Significant main effects or interactions were followed by pairwise comparisons based on estimated marginal means.

To complement Western blot analysis, HIF1A levels were also quantified using an ELISA‑based approach. ELISA measurements were performed both on lung samples (from 23 mice in total) and spleen samples (from 21 mice in total). HIF1A ELISA was conducted using the Mouse HIF‑1α ELISA Kit (Abcam, #ab275103) following the manufacturer’s protocol. Briefly, 20–40 mg of tissue was homogenized following the same steps previously described. For ELISA, instead of RIPA buffer, we used 700 µL of the PTR buffer provided in the kit and centrifuged at 13,000×*g* for 5 min at 4 °C. Supernatants were collected, and protein concentrations were determined using a BCA Protein Assay Kit (Thermo Fisher Scientific, #23225). Lysates were normalized by diluting all samples in PTR buffer to match the lowest protein concentration. For each well, 40 µg of adjusted lysate in a 50 µL volume was loaded onto the ELISA plate. Absorbance at 450 nm was measured using a GloMax Discover plate reader. Of note, all values fell within the linear range of the standard curve. For statistical analysis, we used a linear mixed‑effects model to test the effects of Age Group (Adult vs. Old), Condition (Unt, IH, IH Recovery), and their interaction on HIF‑1α levels.

### DNA extraction and sample preparation

DNA was extracted from heart, spleen, and lung tissues using the Agilent kit (Catalog #200600), yielding an average concentration of 1.02 µg/µl. Samples were diluted to 25 ng/µl and processed using the HorvathMammalMethylChip320 Infinium methylation array (~320,000 CpGs)^113^ at the Epigenetic Clock Foundation (Torrance, USA). A total of 108 samples were analyzed, stratified by age, condition, and tissue type (*n* = 6 per group).

Raw DNA methylation data were processed using the SeSAMe pipeline, which is optimized for mammalian methylation arrays^114^. This approach includes background correction and dye-bias normalization to minimize technical artifacts. Sample-level quality control was performed using hierarchical clustering based on pairwise Pearson correlation of methylation beta values. Distances were defined as 1 − correlation, and clustering was performed using average linkage. Clusters were identified using the cutreeStatic function (cut height = 0.015, minSize = 2). Samples primarily clustered by tissue type, with additional separation by age group. Outlier samples were identified based on their separation from the main clusters and removed from downstream analyses. Specifically, samples from one mouse did not cluster with their corresponding tissue groups and were excluded. In addition, one lung sample clustered with spleen samples and was reannotated as spleen based on clustering and tissue prediction. In total, six samples were removed, resulting in 102 samples retained for downstream analysis.

### Epigenetic clock analysis

To focus on epigenetic aging changes close to genes conserved across mammals, we employed the universal pan-mammalian Universal Clock 3 (UC3), also known as the universal log-linear age clock, that enables us to predict age in pan-tissue across a variety of mammals. This clock was established based on data from the mammalian 40 K array, which covers CpGs located within conserved genomic regions^10^. To provide an additional comparison, we repeated the analysis using two alternative clocks: (1) the Universal Clock 2, which estimates relative age across species (age divided by maximum lifespan) and (2) the mouse pan-tissue clock (mouse DNAmAge clock)^28^.

These epigenetic clocks have been widely adopted and used in the literature. Both universal clocks have been extensively validated in nearly 10,000 samples across 59 tissue types and 128 species, including mice. These clocks are implemented in the dedicated R package MammalMethylClock^115^.

### Epigenome-wide association studies (EWAS)

We performed a tissue-specific EWAS to identify the CpG sites in which DNA methylation changes mimicked the reversible effects of hypoxia in old and adult mice. We focused on two distinct methylation patterns: an A-shaped pattern where a CpG gained methylation during IH and finally lost methylation during recovery; and a U-shaped pattern where a CpG lost methylation during IH and finally regained methylation during recovery.

To identify these sites, we used a linear regression model with DNA methylation levels (beta values) as the dependent variable. We included a variable encoding the IH, recovery, and control groups versus the IH group. Specifically, the IH group was coded as one, while the control and recovery groups were coded as zeros. Consequently, a positive effect size indicates an A-shape pattern (a CpG that gains methylation in response to hypoxia and recovers in the recovery phase). Conversely, a negative effect size indicates a U-shape pattern, a CpG that loses methylation in response to hypoxia and regains baseline methylation levels in the recovery phase. This modeling approach is particularly robust in capturing reversible effects because our experimental design utilized cohorts of the same sex and age within each group (adult and old), thereby minimizing biological heterogeneity and ensuring that the identified epigenetic shifts are specifically attributable to the hypoxia-recovery cycle.

To characterize these reversible effects, we selected only the top, most significant 500 CpGs for both the A- and U-shape patterns. By restricting our analysis to this top-tier subset, we ensure that the selected sites robustly exhibit the underlying A-shaped or U-shaped patterns, particularly given that our analysis relies on straightforward univariate linear regression models. Furthermore, we applied this conservative 500-CpG threshold consistently across all tissues and age groups in our tissue- and age-specific EWAS to ensure a uniform and comparable stringency throughout the study. Across all analyses, this threshold maintained a significance level of *P* < 0.02; for instance, in the old lung EWAS, all top 500 CpGs reached a significance of *P* < 0.001. To confirm that this model accurately captured the reversible effects of hypoxia, we visualized the mean methylation levels of the top 20 CpGs against experimental conditions. Subsequent analyses, including biological pathways and motif-based annotations, were based on these prioritized 500 A-shape and 500 U-shape CpGs, ranked by EWAS *P* values.

### Genomic regions enrichment of annotations tool (GREAT) analysis

We conducted a GREAT analysis^116^ to identify biological pathways and functional annotations enriched in genomic regions containing CpGs that exhibited reversible hypoxia-associated methylation changes. To guarantee adequate statistical power, we used the top 500 CpGs with positive and negative associations from our EWAS. We implemented canonical foreground (background) hypergeometric tests to account for the number of CpGs per gene and gene size bias inherent to the 320 K mammalian methylation array. The foreground was defined by the genomic regions of the top 500 CpGs, while the background comprised all genomic regions represented on the array. The analysis was performed using the rGREAT package in R.

We set the GREAT analysis parameters to match the pipeline used in the studies of the Mammalian Methylation Consortium, therefore matching the hg19 human genome assembly. To translate findings from human chromatin states to mice, we mapped the 320,000 CpGs from the mm10 (mouse) genome to the hg19 (human) genome using the R liftOver package. The proximal regions were defined as 5.0 kb upstream and 1.0 kb downstream, and distal regions extending up to 50 kb, consistent with our previous work^8^.

We incorporated annotations from 13 established pathway and ontology databases, including Mouse Phenotype, BioCyc Pathway, Disease Ontology, Gene Ontology (Biological Process and Molecular Function), Human Phenotype Ontology, MGI Expression: Detected, and Panther Pathways. In addition, we included five MSigDB collections: Pathway, Perturbation, Predicted Promoter Motifs, Immunologic Signatures, and Oncogenic Signatures. To focus on biologically meaningful enrichments, we refined our results based on several criteria. Gene sets were only considered if they had a minimum of three overlapping genomic regions and five overlapping genes. Furthermore, we applied a filter to avoid overly broad gene sets by calculating an ‘annotation region ratio’: the number of genomic regions in the annotated gene set divided by the total number in the background. Only enrichments for gene sets with a ratio less than 0.35 were considered.

### Hypergeometric optimization of motif EnRichment (HOMER) analysis

We applied HOMER v5.1^117^ to perform genomic region motif analysis and identify enriched transcription factor (TF) binding sites close to CpGs whose methylation underwent reversible hypoxia effects. As part of the HOMER settings, we applied guanine-cytosine (GC)-content normalization. When this option is enabled, HOMER also performs an automatic normalization of sequence bias based on lower-order k-mers. Specifically, the algorithm adjusts the weights of background sequences according to differences in short oligonucleotide frequencies between target and background regions. In our analysis, we used the default setting (nlen = 3), which normalizes sequence composition up to 3-mers. This additional step further reduces bias introduced by local sequence composition when evaluating transcription factor motif enrichment. The target regions were defined as the top 500 CpGs with positive and negative associations from our EWAS results. The background regions were based on the genomic regions across our 320k mouse array. The analysis was performed using the mm10 genome with default options, including a region size of 200 bp for motif finding. Normalization was based on GC content, two mismatches were allowed during the global optimization phase, and motif scoring was based on unadjusted hypergeometric *P* values. We report the enrichment of known motifs with a *P* value <0.01, as well as de novo motifs that are not flagged as “possible false positive” by HOMER.

### Universal chromatin state analysis

To functionally annotate the CpGs with reversible IH effects, we used a universal ChromHMM chromatin state model^118^. This model, which was trained on over 1000 datasets, provides a single, robust annotation for the human genome (hg19) that is applicable across diverse cell and tissue types. It classifies the genome into 100 distinct states, which are further grouped into 16 major functional categories such as promoters and enhancers. Several CpGs overlapped between two chromatin states. A unique chromatin state was successfully assigned to 226,359 CpGs. For each EWAS comparison, we selected the top 500 CpGs with the strongest positive associations and the top 500 with the strongest negative associations for analysis. To assess the overlap of these selected CpGs with the chromatin states, we performed a one-sided hypergeometric analysis to evaluate both enrichment (fold change >1) and depletion (fold change <1). The analysis was restricted to the 98 chromatin states containing at least 100 CpGs.

While the human genome chromatin state annotation was used for comparison with pan-mammalian EWAS of aging, we also performed a sensitivity check using an analogous chromatin state annotation for the mouse genome^119^. This mouse full-stack model was developed by Vu et al. using 901 datasets assaying 14 chromatin marks across 26 cell and tissue types. As noted, the mouse chromatin states, which also include 100 states, were analogously defined with human states. We used this resource to verify the robustness of our functional findings within a mouse-specific genomic context.

### Polycomb repressive complex 2 (PRC2) examination

PRC2 binding sites were annotated based on the overlap region with H3K27me3 modification (a hallmark of PRC2 activity). These regions were identified from publicly available ENCODE H3K27me3 ChIP-seq data derived from multiple mouse tissue types. The reference datasets were primarily generated from mice at embryonic day 14 (E14) and 8 weeks of age, spanning multiple tissue types including brain, heart, kidney, liver, small intestine, spleen, testes, and thymus. Broad PRC2/H3K27me3 domains are largely established during development and are highly conserved across different ages and cell types^120,121^. We aggregated data across all available ENCODE experiments and multiple tissue types to provide a robust and comprehensive map of canonical PRC2 target regions. Furthermore, age-associated changes in H3K27me3 typically involve quantitative shifts in modification levels at existing targets rather than the wholesale gain or loss of entirely new PRC2 domains^122^. Therefore, these defined regions provide a functional genomic framework for PRC2 occupancy that can be applied to our mouse data across different age groups.

Of our 320,000-probe array, 4424 CpGs were successfully annotated within these defined PRC2 binding regions. Analysis of PRC2 annotation for hypoxia EWAS was performed using the same hypergeometric analysis methods previously described for the universal chromatin state annotation.

### Longitudinal DNA methylation data in hypobaric hypoxia during human acclimatization at Mt. Chacaltaya (5260 meters)

We used publicly available human blood DNA methylation data from the AltitudeOmics project (GEO accession GSE105123) in which young individuals (*n* = 19; 11 males, 8 females; mean age 20.8 ± 1.4 years) underwent baseline testing near sea level (Eugene, Oregon, USA; 130 m) and then again after rapid ascent to high altitude (Mt. Chacaltaya, Bolivia; 5260 m), where they remained for 16 days^82^. Our analysis focused on five consecutive time points: (I) baseline (near sea level), then (II) at noon once reaching high altitude at 5260 m, (III) after 1 h (day 1, for assessing the quick acclimation) and then two subsequent measurements (Day 7 and Day 16), yielding 88 samples in total.

DNA methylation arrays were profiled using the Illumina Infinium HumanMethylation450 BeadChip and processed in R using the minfi package with Noob normalization. To ensure consistency with our prior murine experiment, we analyzed human blood methylation data by the Universal pan-mammalian Clocks 2 and 3. Estimates for these Universal Clocks were derived using a conversion algorithm from Infinium 450k array data, as previously described^10^. While the Universal Clocks provided cross-species consistency, we also performed a comprehensive analysis using clocks optimized for human samples, such as DNAmGrimAge2^123^, which is predictive of mortality risk. We also included its underlying components that estimate plasma protein levels, e.g., DNAmADM (which estimates ADM levels) and DNAmB2M (which estimates B2M levels). Given the unresolved debate regarding the best DNAm clocks or biomarkers, we also conducted an exploratory analysis of 35 DNAm-based biomarkers most used in the literature (Supplementary Data 61). This set included first-generation human clocks such as Horvath’s pan-tissue clock (DNAmAge)^11^ and next-generation clocks such as the DNAmGrimAge2 clock^123^ and the DunedinPACE clock^7^. The principal component (PC) versions of these clocks were included where available^124^. All clock and DNAm biomarker estimates were computed in R. The DunedinPACE estimates were calculated using the official package.

A linear mixed-effects (LME) model was fitted for each of the 35 biomarkers (as the dependent variable) on the five time points, treated as a categorical variable. Baseline was set as the reference, and the models were adjusted for sex, BMI, and chronological age as fixed effects.

For the EWAS, we used the same LME model framework, except that the five time points were encoded as a continuous variable representing duration at high altitude (0, 1.5, 1.54, 7, and 16 days), rather than as a categorical variable. This model tested, for each CpG, whether the DNAm level was associated with increasing duration of high-altitude exposure.

## Supplementary information

Supplementary information

Supplementary information

## Data Availability

All methylation data will be posted in Gene Expression Omnibus. The mammalian methylation array is available through the non-profit Epigenetic Clock Development Foundation (Torrance, USA).

## Code availability

All software scripts are available^115^— MammalMethylClock R package. The R scripts used for generating the figures are available upon request to Steve Horvath (shorvath@mednet.ucla.edu) and Luigi Ferrucci (ferruccilu@grc.nia.nih.gov),
