# Supplementary material for: Intermittent hypoxia induces reversible epigenetic age acceleration in old mice
Source: NPJ Aging. 2026 Jun 23;12(1):145. doi: 10.1038/s41514-026-00425-2 (PMC13672789; doi:10.1038/s41514-026-00425-2)
Supplement: Supplementary file 1 [file 41514_2026_425_MOESM1_ESM.docx]

**SUPPLEMENTARY FIGURES**

**Supplementary Figure 1. Age-effect of HIF-1α expression at baseline. a)** Western blot analysis of lung tissue from adult and old mice (n=8 per group). Lighter blue bars represent adult mice, and darker blue bars represent old mice. The lower panel shows densitometric quantification of HIF-1α normalized to Actin (y-axis: fold change). **b)** HIF-1α expression after treatments: Western blot analysis at study endpoint for adult (left) and old (right) mice. Each gel represents triplicate samples. Lower panel shows densitometric quantification of HIF-1α normalized to Actin and expressed relative to the Control condition**. c)** Lung ELISA quantification of HIF‑1α protein levels in adult and old mice. Box plots display HIF‑1α concentrations (y‑axis, pg/mL) in lung tissue across three experimental conditions (Control, IH, IH Recovery) for each age group. Individual data points are overlaid on the boxes to show the distribution of values within each group. **d)** Spleen ELISA quantification of HIF‑1α protein levels in adult and old mice. Box plots display HIF‑1α concentrations (y‑axis, pg/mL) in spleen tissue across the same three experimental conditions (Control, IH, IH Recovery) for each age group, with individual data points overlaid to illustrate within‑group variability.

**Supplementary Figure 2. Uncropped Western blot membranes corresponding to Supplementary Figure 1. a)** Western blots of HIF-1α and Actin in lung tissue from adult and old control mice (no IH exposure). Blots were run with n = 4 per age group, with molecular weights hand-marked directly on the membranes. Full membranes are shown without cropping or modification. **b, c)** Western blots of HIF-1α and Actin in lung tissue from adult (b) and old (c) mice across all experimental groups (control, IH, and IH recovery; n = 3 per group, run in triplicate). Molecular weights are indicated digitally.

**Supplementary Figure 3. Correlation heatmaps of epigenetic clocks across tissues and conditions.** Heatmap for **a)** adult and **b)** old mice show pairwise correlations between epigenetic clock estimates (rows) within each tissue-condition group (columns) for a) adult mice and b) old mice. Rows correspond to clock pairs: universal clock 3 vs universal clock 2, universal clock 3 vs mouse DNAmAge clock, and universal clock 2 vs DNAmAge clock (computed use Epigenetic age values expressed in years). Values represent Pearson correlation coefficients (r), and the color scale is displayed as a horizontal legend with blue = negative, white = 0, and red = positive (−1 to +1 from left to right). No clustering was applied so the specified column order is preserved. Coefficients are Pearson correlations.

**Supplementary Figure 4. Hierarchical clustering of mouse DNA methylation samples.** Hierarchical clustering was performed on DNA methylation array data from 108 mouse samples. Pairwise Pearson correlations of beta values were computed, and distances were defined as 1 − correlation. Clustering was performed in R using average linkage, and the dendrogram with sample annotations was visualized using the WGCNA function plotDendroAndColors. Clusters were defined using cutreeStatic (cutHeight = 0.015, minSize = 2). The color bars below the dendrogram indicate cluster assignment (grey indicates samples not assigned to a cluster), outliers (red), original tissue annotation, updated tissue annotation (based on predicted tissue identity), age group (dark green: old; light green: young), and experimental condition (grey: control; red: intermittent hypoxia (IH); orange: IH recovery). Clustering revealed that samples primarily group by tissue type, with secondary separation by age. Four outlier samples (marked with asterisks) were identified, including a lung sample from mouse GW29_703. As noted, other tissues from this mouse (heart and spleen) did not cluster with their respective tissue groups, and the entire mouse was excluded from downstream analyses, resulting in 102 samples. In addition, one lung sample from mouse GW29_029 clustered with spleen samples and was also predicted as spleen tissue by our in-house algorithm; this sample was therefore reannotated as spleen.

**Supplementary Figure 5. Top CpGs exhibiting A-shape effects in the lung EWAS in old mice.** Panels a–t show the DNA methylation profiles for the top 20 CpG sites identified in the lung EWAS exhibiting an "A-shape" association. The genome assembly and coordinates are based on **mm10**. Individual data points are overlaid on notched boxplots; the central horizontal line and blue numerical labels represent the median, while the box indicates the interquartile range (IQR).Each panel title specifies the annotations of the CpG including chromosome, proximal gene symbol, genomic region, and distances to the Transcription Start Site (in kb), where negative values denote locations upstream of the TSS. The genome assembly is based on mm10. Inter_Up denotes an intergenic region located upstream (5') of the TSS, while Inter_Down refers to an intergenic region located downstream (3') of the gene end.

**Supplementary Figure 6. Top CpGs exhibiting U-shape effects in the lung EWAS in old mice.** Panels a–t show the DNA methylation profiles for the top 20 CpG sites identified in the lung EWAS exhibiting an "U-shape" association. The genome assembly and coordinates are based on **mm10**. Individual data points are overlaid on notched boxplots; the central horizontal line and blue numerical labels represent the median, while the box indicates the interquartile range (IQR).Each panel title specifies the annotations of the CpG including chromosome, proximal gene symbol, genomic region, and distances to the Transcription Start Site (in kb), where negative values denote locations upstream of the TSS. The genome assembly is based on mm10. Inter_Up denotes an intergenic region located upstream (5') of the TSS, while Inter_Down refers to an intergenic region located downstream (3') of the gene end.

**Supplementary Figure 7. Top CpGs exhibiting A-shape effects in the spleen EWAS in old mice.** Panels a–t show the DNA methylation profiles for the top 20 CpG sites identified in the spleen EWAS exhibiting an "A-shape" association. The genome assembly and coordinates are based on **mm10**. Individual data points are overlaid on notched boxplots; the central horizontal line and blue numerical labels represent the median, while the box indicates the interquartile range (IQR).Each panel title specifies the annotations of the CpG including chromosome, proximal gene symbol, genomic region, and distances to the Transcription Start Site (in kb), where negative values denote locations upstream of the TSS. The genome assembly is based on mm10. Inter_Up denotes an intergenic region located upstream (5') of the TSS, while Inter_Down refers to an intergenic region located downstream (3') of the gene end.

**Supplementary Figure 8. Top CpGs exhibiting U-shape effects in the spleen EWAS in old mice.** Panels a–t show the DNA methylation profiles for the top 20 CpG sites identified in the spleen EWAS exhibiting an "U-shape" association. The genome assembly and coordinates are based on **mm10**. Individual data points are overlaid on notched boxplots; the central horizontal line and blue numerical labels represent the median, while the box indicates the interquartile range (IQR).Each panel title specifies the annotations of the CpG including chromosome, proximal gene symbol, genomic region, and distances to the Transcription Start Site (in kb), where negative values denote locations upstream of the TSS. The genome assembly is based on mm10. Inter_Up denotes an intergenic region located upstream (5') of the TSS, while Inter_Down refers to an intergenic region located downstream (3') of the gene end.

**Supplementary Figure 9. Top CpGs exhibiting A-shape effects in the heart EWAS in old mice.** Panels a–t show the DNA methylation profiles for the top 20 CpG sites identified in the heart EWAS exhibiting an "A-shape" association. The genome assembly and coordinates are based on **mm10**. Individual data points are overlaid on notched boxplots; the central horizontal line and blue numerical labels represent the median, while the box indicates the interquartile range (IQR).Each panel title specifies the annotations of the CpG including chromosome, proximal gene symbol, genomic region, and distances to the Transcription Start Site (in kb), where negative values denote locations upstream of the TSS. The genome assembly is based on mm10. Inter_Up denotes an intergenic region located upstream (5') of the TSS, while Inter_Down refers to an intergenic region located downstream (3') of the gene end.

**Supplementary Figure 10. Top CpGs exhibiting U-shape effects in the heart EWAS in old mice.** Panels a–t show the DNA methylation profiles for the top 20 CpG sites identified in the heart EWAS exhibiting an "U-shape" association. The genome assembly and coordinates are based on **mm10**. Individual data points are overlaid on notched boxplots; the central horizontal line and blue numerical labels represent the median, while the box indicates the interquartile range (IQR).Each panel title specifies the annotations of the CpG including chromosome, proximal gene symbol, genomic region, and distances to the Transcription Start Site (in kb), where negative values denote locations upstream of the TSS. The genome assembly is based on mm10. Inter_Up denotes an intergenic region located upstream (5') of the TSS, while Inter_Down refers to an intergenic region located downstream (3') of the gene end.

**Supplementary Figure 11. Adult mice Epigenome-wide association studies (EWAS) in lungs, spleen, and heart.** **a–c)** Miami plots for adult **a)** lung, **b)** spleen, and **c)** heart. The upper half depicts CpGs fitting a reversible A-shape model (hypermethylation during IH), and the lower half depicts CpGs that fit a reversible U-shape model (hypomethylation during IH). A red dashed line marks the threshold for significance (p < 1e⁻⁵); CpGs surpassing this threshold are highlighted in red (A-shape) or blue (U-shape). **d–f)** Top differentially methylated CpGs exhibiting reversible hypoxia effects in adult mice (“Italian Moka” plots) for **d)** lung, **e)** spleen, and **f)** heart. Panels show average methylation at the top three CpGs per tissue. CpGs with positive (A-shape) effects are displayed in the left column; CpGs with negative (U-shape) effects are displayed in the right column. Significance of methylation levels in y-axis is represented by the −log₁₀(p-value) as well as the CpG sites across chromosomal position (mm10) for Control (dark gray), IH (red), and IH Recovery (orange).

**Supplementary Figure 12. Top CpGs exhibiting A-shape effects in the lung EWAS in adult mice.** Panels a–t show the DNA methylation profiles for the top 20 CpG sites identified in the lung EWAS exhibiting an "A-shape" association. The genome assembly and coordinates are based on mm10. Individual data points are overlaid on notched boxplots; the central horizontal line and blue numerical labels represent the median, while the box indicates the interquartile range (IQR).Each panel title specifies the annotations of the CpG including chromosome, proximal gene symbol, genomic region, and distances to the Transcription Start Site (in kb), where negative values denote locations upstream of the TSS. The genome assembly is based on mm10. Inter_Up denotes an intergenic region located upstream (5') of the TSS, while Inter_Down refers to an intergenic region located downstream (3') of the gene end.

**Supplementary Figure 13. Top CpGs exhibiting U-shape effects in the lung EWAS in adult mice.** Panels a–t show the DNA methylation profiles for the top 20 CpG sites identified in the lung EWAS exhibiting an "U-shape" association. The genome assembly and coordinates are based on **mm10**. Individual data points are overlaid on notched boxplots; the central horizontal line and blue numerical labels represent the median, while the box indicates the interquartile range (IQR).Each panel title specifies the annotations of the CpG including chromosome, proximal gene symbol, genomic region, and distances to the Transcription Start Site (in kb), where negative values denote locations upstream of the TSS. The genome assembly is based on mm10. Inter_Up denotes an intergenic region located upstream (5') of the TSS, while Inter_Down refers to an intergenic region located downstream (3') of the gene end.

**Supplementary Figure 14. Top CpGs exhibiting A-shape effects in the spleen EWAS in adult mice.** Panels a–t show the DNA methylation profiles for the top 20 CpG sites identified in the spleen EWAS exhibiting an "A-shape" association. The genome assembly and coordinates are based on **mm10**. Individual data points are overlaid on notched boxplots; the central horizontal line and blue numerical labels represent the median, while the box indicates the interquartile range (IQR).Each panel title specifies the annotations of the CpG including chromosome, proximal gene symbol, genomic region, and distances to the Transcription Start Site (in kb), where negative values denote locations upstream of the TSS. The genome assembly is based on mm10. Inter_Up denotes an intergenic region located upstream (5') of the TSS, while Inter_Down refers to an intergenic region located downstream (3') of the gene end.

**Supplementary Figure 15. Top CpGs exhibiting U-shape effects in the spleen EWAS in adult mice.** Panels a–t show the DNA methylation profiles for the top 20 CpG sites identified in the spleen EWAS exhibiting an "U-shape" association. The genome assembly and coordinates are based on **mm10**. Individual data points are overlaid on notched boxplots; the central horizontal line and blue numerical labels represent the median, while the box indicates the interquartile range (IQR).Each panel title specifies the annotations of the CpG including chromosome, proximal gene symbol, genomic region, and distances to the Transcription Start Site (in kb), where negative values denote locations upstream of the TSS. The genome assembly is based on mm10. Inter_Up denotes an intergenic region located upstream (5') of the TSS, while Inter_Down refers to an intergenic region located downstream (3') of the gene end.

**Supplementary Figure 16. Top CpGs exhibiting A-shape effects in the heart EWAS in adult mice.** Panels a–t show the DNA methylation profiles for the top 20 CpG sites identified in the heart EWAS exhibiting an "A-shape" association. The genome assembly and coordinates are based on **mm10**. Individual data points are overlaid on notched boxplots; the central horizontal line and blue numerical labels represent the median, while the box indicates the interquartile range (IQR).Each panel title specifies the annotations of the CpG including chromosome, proximal gene symbol, genomic region, and distances to the Transcription Start Site (in kb), where negative values denote locations upstream of the TSS. The genome assembly is based on mm10. Inter_Up denotes an intergenic region located upstream (5') of the TSS, while Inter_Down refers to an intergenic region located downstream (3') of the gene end.

**Supplementary Figure 17. Top CpGs exhibiting U-shape effects in the heart EWAS in old mice.** Panels a–t show the DNA methylation profiles for the top 20 CpG sites identified in the heart EWAS exhibiting an "U-shape" association. The genome assembly and coordinates are based on **mm10**. Individual data points are overlaid on notched boxplots; the central horizontal line and blue numerical labels represent the median, while the box indicates the interquartile range (IQR).Each panel title specifies the annotations of the CpG including chromosome, proximal gene symbol, genomic region, and distances to the Transcription Start Site (in kb), where negative values denote locations upstream of the TSS. The genome assembly is based on mm10. Inter_Up denotes an intergenic region located upstream (5') of the TSS, while Inter_Down refers to an intergenic region located downstream (3') of the gene end.

**Supplementary Figure 18. Shared differentially methylated CpG sites across lung, spleen, and heart tissues in adult and old mice.** a) Hypermethylated CpGs. b) Hypomethylated CpGs. Each tissue is represented by two columns, with adult samples on the left and old samples on the right. Shown are genes nearest to CpG sites differentially methylated in at least three of the six tissue-age combinations, selected from the top 500 EWAS-identified loci. Dot color indicates Z-score; dot size reflects statistical significance (−log₁₀(p-value)).

**Supplementary Figure 19. Correlation between all EWAS Z-scores for intermittent hypoxia (IH) effects old versus adult mice.** Panels are organized by tissue for **a)** lung, **b)** spleen and **c)** heart); the x-axes show Z-scores in old mice, and the y-axes show Z-scores in adult mice. Each point represents a CpG assayed on the mouse 320k array and mapped to the mm10 genome. Pearson’s correlation coefficient (r) and its two-sided P-value are reported in each panel. The 10 CpGs with the largest positive and negative products of Z-scores are labeled: positively associated sites (red) gain methylation during IH and lose it during recovery, whereas negatively associated sites (blue) lose methylation during IH and regain it during recovery.

**Supplementary Figure 20. Adult mice matching old mice functional enrichment (GREAT analysis) of reversible CpGs.** For comparison, the pathways represented match those shown in Figure 4 (old mice). The figure is structured into six panels as for Figure 4: three for methylation gain and three for methylation loss, each subdivided by tissue (heart, lung, spleen EWAS, respectively). The bar plots in the first column display the total number of genes within each enriched set, adjusted for background. Each cell in the main panels reports both the fold change and the corresponding unadjusted hypergeometric p-value. The color gradient of each cell corresponds to the −log10(p), with the scale matching that used in Figure 4. A column color band on the right denotes the ontology source of each gene set. Pathways were further highlighted into three macro-categories: hypoxia/aging-related (red labels), developmental/prenatal-related (blue labels), and cancer-related (purple labels).

**Supplementary Figure 21. IH-responsive CpGs are mostly localized to Bivalent and Polycomb chromatin states in old mice.** Heatmaps display the results of a hypergeometric overlap analysis of tissue-specific EWAS conducted in old mice, showing that CpGs with reversible methylation changes localize to specific chromatin states. The analysis was performed for the top 500 CpGs with a reversible -positive- methylation gain or A-shape (left panels) or a reversible -negative- methylation loss or U-shape (right panels) association from EWAS. Rows of the heatmaps represent selective universal chromatin states and PRC2 binding regions. Bar plots to the left of each heatmap indicate the proportion of CpGs located within PRC2 binding regions. The y-axis lists the chromatin state or PRC2 category, along with the total number of CpGs in parentheses. Each heatmap cell reports the FC and the corresponding unadjusted hypergeometric P-value. The color gradient represents the signed −log10-transformed unadjusted hypergeometric P-value, where red indicates enrichment (FC > 1) and blue indicates depletion (FC < 1).

**Supplementary Figure 22. Chromatin state and Polycomb Repressive Complex 2 (PRC2) annotations for mammalian EWAS of age.** Heatmap reproduced from our previous mammalian study (Lu et al., 2023, see Figure 4h), shows the annotation results for EWAS of age across 65 mammalian species and all tissue types (10,771 samples). The heatmap visualizes a hypergeometric overlap analysis between age-related CpGs (columns) and annotations for 1) selective universal chromatin states and 2) PRC2 binding sites. The first column contains a bar plot showing the proportion of CpGs that are known to be bound by PRC2, with a range from zero to one. Each cell in the heatmap reports the Odds Ratio (OR) and the unadjusted P-value from the hypergeometric test. The analysis includes the top 1,000 CpGs with methylation that increased or decreased with age from a meta-EWAS of all tissues, as well as individual analyses for blood, skin, liver, muscle, brain, and cerebral cortex. The heatmap's color gradient is based on the −log10(P−value) multiplied by the sign of the OR when the OR is greater than one. Red colors indicate an OR greater than one (enrichment), while blue colors indicate an OR less than one (depletion).

**Supplementary Figure 23. IH-responsive CpGs localization to chromatin states in adult mice.** Heatmaps display the results of a hypergeometric overlap analysis of tissue-specific EWAS conducted in adult mice, showing that CpGs with reversible methylation changes localize to specific chromatin states. The analysis was performed for the top 500 CpGs with a reversible -positive- methylation gain or A-shape (left panels) or a reversible -negative- methylation loss or U-shape (right panels) association from EWAS. Rows of the heatmaps represent selective universal chromatin states and PRC2 binding regions. Bar plots to the left of each heatmap indicate the proportion of CpGs located within PRC2 binding regions. The y-axis lists the chromatin state or PRC2 category, along with the total number of CpGs in parentheses. Each heatmap cell reports the FC and the corresponding unadjusted hypergeometric P-value. The color gradient represents the signed −log10-transformed unadjusted hypergeometric P-value, where red indicates enrichment (FC > 1) and blue indicates depletion (FC < 1).

**Supplementary Figure 24. Reversible PRC2 methylation changes in old mice exposed to intermittent hypoxia.** This figure shows the average methylation levels at PRC2 binding sites (y-axis) across three treatment groups (x-axis): Control, IH, and IH+Recovery (IH_Recovery). A combined group (Control + IH_Recovery) is also displayed and labeled not_IH. The plots are stratified by age group (adult and old) and tissue type. For each panel, a student’s t-test was performed to compare methylation levels among the three treatment groups as well as IH versus not_IH.

**Supplementary Figure 25. Mouse chromatin state annotation for EWAS results in old mice.** Heatmaps display the results of a hypergeometric overlap analysis of tissue-specific EWAS conducted in old mice with 100 mouse chromatin states. The analysis was performed for the top 500 CpGs with a reversible positive methylation gain (A-shape, left panels) or a reversible negative methylation loss (U-shape, right panels) associated with IH. Rows of the heatmaps represent a total of 100 universal chromatin states in mouse genome. Bar plots to the left of each heatmap indicate the proportion of CpGs located within PRC2 binding regions. The y-axis lists the chromatin state or PRC2 category, along with the total number of CpGs in parentheses. Each heatmap cell reports the FC and the corresponding unadjusted hypergeometric P-value, provided P-value<0.05. The color gradient represents the signed -log10-transformed unadjusted hypergeometric P-value, where red indicates enrichment (FC > 1) and blue indicates depletion (FC < 1).

**Supplementary Figure 26. Mouse chromatin state annotation for EWAS results in adult mice.** Heatmaps display the results of a hypergeometric overlap analysis of tissue-specific EWAS conducted in old mice with 100 mouse chromatin states. The analysis was performed for the top 500 CpGs with a reversible positive methylation gain (A-shape, left panels) or a reversible negative methylation loss (U-shape, right panels) associated with IH. Rows of the heatmaps represent a total of 100 universal chromatin states in mouse genome. Bar plots to the left of each heatmap indicate the proportion of CpGs located within PRC2 binding regions. The y-axis lists the chromatin state or PRC2 category, along with the total number of CpGs in parentheses. Each heatmap cell reports the FC and the corresponding unadjusted hypergeometric P-value, provided P-value<0.05. The color gradient represents the signed -log10-transformed unadjusted hypergeometric P-value, where red indicates enrichment (FC > 1) and blue indicates depletion (FC < 1).

**Supplementary Figure 27. Transcription factors enriched across multiple tissues and age groups.** This figure displays a selective set of transcription factors (TFs) significantly enriched in EWAs of reversible hypoxia. The enrichment analysis was performed using HOMER for the top 500 CpGs with positive and negative associations from the six tissue and age specific EWASs, yielding a total of 12 sets of results. A TF was included in this figure if it was significantly identified from the known motif analysis (with an unadjusted hypergeometric p<0.01 and FDR < 0.1) and appeared in four of the 12 sets of results. No TF appeared in more than four sets. The x-axis represents the −log10 unadjusted P-value, while the y-axis lists the motif name. The plot is stratified by age, tissue, and the sign of association (positive A-shape in red or negative U-shape in blue).

**Supplementary Figure 28. Individual Age Acceleration Trajectories during Hypobaric Hypoxia.**

**a–c)** Longitudinal trajectories of Epigenetic Age Acceleration (AgeAccel) estimated by **a)** DNAmGrimAge2, **b)**UniversalClock2, and **c)** UniversalClock3. Y-axes represent AgeAccel measurements (years). X-axes display five sequential time points: baseline (130m altitude [ALT]) and high-altitude exposure (5260m) at Day 1 (Noon and 1 PM), Day 7, and Day 16. The trajectories of individual participants are connected by colored dashed lines (spaghetti plot). The mean AgeAccel estimate across all participants for each time point is overlaid in red.

**Supplementary Figure 29. PRC2 methylation during human acclimatization to hypobaric hypoxia.**  (a) Mean PRC2 methylation was computed longitudinally across five time points. The x-axis represents the study timeline, including baseline near sea level (130 m), followed by high-altitude exposure (5260 m) at Day 1 (noon and 1 PM), Day 7, and Day 16. Mean PRC2 methylation levels at each time point are shown in red. The title in panel a indicates the range of mean PRC2 methylation levels. (b) The table shows the results of a linear mixed-effects (LME) model testing the association between mean PRC2 methylation and time point (relative to baseline), adjusted for sex, BMI, and chronological age. Reported P values are unadjusted. (c) The panel shows individual age-adjusted PRC2 methylation trajectories during hypobaric hypoxia. The x-axis displays the five sequential time points, and the y-axis represents age-adjusted PRC2 methylation. Individual participant trajectories are connected by colored dashed lines (spaghetti plot). The mean age-adjusted PRC2 methylation levels across all participants at each time point are shown in red.

**Supplementary Figure 30. Manhattan plot of blood EWAS for human acclimatization to hypobaric hypoxia.** The figure displays the EWAS results for human acclimatization to hypobaric hypoxia. A linear mixed-effects model was applied to longitudinal blood DNAm data profiled at five consecutive time points: day 0 (baseline near sea level, 130 m) and four subsequent time points during exposure to high altitude (5260 m). The model tested the association between DNAm level (dependent variable) and duration of high-altitude exposure (0, 1.5, 1.54, 7, and 16 days as the independent variable), while adjusting for age, sex, and BMI. The upper panel shows CpGs that gain methylation with increasing duration at high altitude, whereas the lower panel shows CpGs that lose methylation. The red dashed line indicates P < 1.0e-05, and CpGs surpassing P < 1.0e-07 are highlighted in red (upper panel) or blue (lower panel). The top 20 CpGs in each panel are annotated by their proximal genes. Y-axes show statistical significance as -log10(P value).

**Supplementary Fig. 31: Chromatin state annotation for blood EWAS of human acclimatization to hypobaric hypoxia (top 10,000 CpGs).** The heatmaps show the results of a hypergeometric overlap analysis between the blood EWAS of human acclimatization to hypobaric hypoxia and (1) 100 universal chromatin states and (2) PRC2 binding sites. The analysis was performed using the top 10,000 CpGs with either increasing or decreasing DNAm with increasing duration at high altitude. Rows represent the 100 universal chromatin states in the human genome and PRC2 binding regions. Bar plots to the left of each heatmap indicate the proportion of CpGs located within each annotated region. The y-axis lists the corresponding chromatin state or PRC2 category, along with the total number of CpGs in parentheses. Each heatmap cell reports the fold change (FC) and the corresponding unadjusted hypergeometric P-value, when P < 0.05. The color gradient represents the signed -log10-transformed unadjusted hypergeometric P-value, where red indicates enrichment (FC > 1) and blue indicates depletion (FC < 1).
